# Patterns of Vestibular Impairment in Bilateral Vestibulopathy and Its Relation to Etiology

**DOI:** 10.3389/fneur.2022.856472

**Published:** 2022-03-21

**Authors:** Lisa van Stiphout, Maksim Pleshkov, Florence Lucieer, Bieke Dobbels, Vergil Mavrodiev, Nils Guinand, Angelica Pérez Fornos, Josine Widdershoven, Michael Strupp, Vincent Van Rompaey, Raymond van de Berg

**Affiliations:** ^1^Division of Balance Disorders, Department of Otorhinolaryngology and Head and Neck Surgery, School for Mental Health and Neuroscience, Maastricht University Medical Center, Maastricht, Netherlands; ^2^Faculty of Physics, Tomsk State University, Tomsk, Russia; ^3^Department of Otorhinolaryngology and Head and Neck Surgery, Faculty of Medicine and Health Sciences, Antwerp University Hospital, University of Antwerp, Antwerp, Belgium; ^4^Department of Neurology and German Center for Vertigo, Ludwig-Maximilians University, Munich, Germany; ^5^Service of Otorhinolaryngology Head and Neck Surgery, Department of Clinical Neurosciences, Geneva University Hospitals, Geneva, Switzerland

**Keywords:** bilateral vestibulopathy, etiology, vestibular implantation, preclinical implantation criteria, Bárány Society diagnostic criteria, vestibular impairment, patterns

## Abstract

**Objective:**

This study aimed to investigate (1) the patterns of vestibular impairment in bilateral vestibulopathy (BVP) and subsequently, the implications regarding patient eligibility for vestibular implantation, and (2) whether this pattern and severity of vestibular impairment is etiology dependent.

**Methods:**

A total of one hundred and seventy-three subjects from three tertiary referral centers in Europe were diagnosed with BVP according to the Bárány Society diagnostic criteria. The subjects underwent vestibular testing such as the caloric test, torsion swing test, video Head Impulse Test (vHIT) in horizontal and vertical planes, and cervical and/or ocular vestibular evoked myogenic potentials (c- and oVEMPs). The etiologies were split into idiopathic, genetic, ototoxicity, infectious, Menière's Disease, (head)trauma, auto-immune, neurodegenerative, congenital, and mixed etiology.

**Results:**

The caloric test and horizontal vHIT more often indicated horizontal semicircular canal impairment than the torsion swing test. The vHIT results showed significantly higher gains for both anterior canals compared with the horizontal and posterior canals (*p* < 0.001). The rates of bilaterally absent oVEMP responses were higher compared to the bilaterally absent cVEMP responses (*p* = 0.010). A total of fifty-four percent of the patients diagnosed with BVP without missing data met all three Bárány Society diagnostic test criteria, whereas 76% of the patients were eligible for implantation according to the vestibular implantation criteria. Regarding etiology, only horizontal vHIT results were significantly lower for trauma, neurodegenerative, and genetic disorders, whereas the horizontal vHIT results were significantly higher for Menière's Disease, infectious and idiopathic BVP. The exploration with hierarchical cluster analysis showed no significant association between etiology and patterns of vestibular impairment.

**Conclusion:**

This study showed that caloric testing and vHIT seem to be more sensitive for measuring vestibular impairment, whereas the torsion swing test is more suited for measuring residual vestibular function. In addition, no striking patterns of vestibular impairment in relation to etiology were found. Nevertheless, it was demonstrated that although the implantation criteria are stricter compared with the Bárány Society diagnostic criteria, still, 76% of patients with BVP were eligible for implantation based on the vestibular test criteria. It is advised to carefully examine every patient for their overall pattern of vestibular impairment in order to make well-informed and personalized therapeutic decisions.

## Introduction

Bilateral vestibulopathy (BVP) is a chronic disease which is characterized by bilaterally reduced or absent vestibular function due to deficits of the vestibular organs, the vestibular nerves, and/or the brain (1–3). Patients typically suffer from imbalance, worsening in the dark and/or on uneven ground, and movement-induced blurred vision (oscillopsia) (4). BVP also leads to additional symptoms such as an increased risk of falling, cognitive deficits, impairment of navigation and spatial memory, autonomic dysfunction, anxiety, and depression (4–11). Consequently, BVP leads to reduced quality of life and imposes a significant socioeconomic burden on society (12–14). BVP appears to be a heterogeneous disorder with various clinical characteristics and multiple identified etiologies, such as ototoxicity (e.g., gentamicin exposure), genetic disorders (e.g., DFNA9), Menière's Disease, infectious causes (e.g., meningitis), neurodegenerative and inherited syndromes (e.g., CANVAS), autoimmunity (e.g., Cogan's syndrome), or trauma (2, 15–23). Nonetheless, the reported percentages of idiopathic BVP vary between 20-75%, indicating that identifying the etiology can be challenging (2, 13, 15, 18, 24).

To date, the prognosis for the recovery of vestibular function is poor and the effective treatment for BVP is missing (18, 25–27). However, different research groups are in the process of developing a clinically applicable vestibular implant that might be able to address at least the major symptoms of BVP (28–35). Despite reaching important milestones in the development of the vestibular implant, many questions remain, and in order to develop a clinically useful device, it is crucial to gain a better understanding of the underlying disease BVP.

So far, it remains unclear which factors contribute to the severity of the vestibular impairment. The current diagnostic criteria for BVP are primarily based on the function of the horizontal semicircular canals (e.g., caloric test, video Head Impulse Test (vHIT), and torsion swing test) (3). However, recent studies have highlighted the varying pattern of impairment of the other vestibular sensors in patients with BVP (i.e., the otolith organs and the anterior and posterior semicircular canals) (24, 36–42). For example, anterior semicircular canal sparing was found in aminoglycoside-related BVP due to bilateral Menière's Disease and in idiopathic BVP (24, 39, 42). Ocular vestibular evoked myogenic potentials, most likely reflecting utricular function, showed to be the most impaired in aminoglycoside-related BVP and the least impaired in BVP due to bilateral Menière's Disease (38). An evidently rare subtype of idiopathic BVP was proposed in which the saccular function was impaired in the presence of normal functioning horizontal semicircular canals (37), while another study showed that horizontal semicircular canal function was more often affected than saccular function in aminoglycoside-related BVP (41).

All studies mentioned above either included small patient groups, retrospectively analyzed the data, did not always include patients with BVP according to the Bárány Society criteria, or investigated only one or two of the vestibular sensors. To date, no studies investigated the pattern of vestibular impairment of all vestibular sensors with relatively large patient groups, while recently published vestibular implantation criteria developed for research settings take all vestibular sensors into consideration. According to these criteria, for instance, all vestibular tests (i.e., caloric test, horizontal and vertical vHIT, and torsion swing test) need to show a significantly impaired function in order to qualify as a vestibular implant candidate (43).

This study provides a description of vestibular function, in a large cohort of patients with BVP diagnosed according to the Bárány Society criteria. The objective was to 1) investigate the patterns of vestibular impairment in BVP in general, and subsequently, the implications regarding patient eligibility for vestibular implantation, and 2) investigate whether the pattern and severity of vestibular impairment depend on the etiology.

## Methods

### Subjects

Study subjects were recruited from three tertiary referral centers in The Netherlands, Belgium, and Germany: The Department of Otorhinolaryngology and Head and Neck surgery from Maastricht University Medical Center (MUMC+, center 1) and Antwerp University Hospital (UZA, center 2), and the Department of Neurology and the German Center for Vertigo and Balance Disorders, Ludwig Maximilians University Munich (LMU, center 3). Enrolled subjects were diagnosed with BVP in accordance with the BVP diagnostic criteria, which included unsteadiness and/or oscillopsia during walking or head movements, and a reduced bithermal caloric response (sum of the bithermal maximal peak slow phase velocity <6°/s bilaterally) and/or a bilaterally reduced horizontal vHIT gain of <0.6, and/or a vestibulo-ocular reflex (VOR) gain <0.1 during torsion swing test at 0.1 Hz (3). In center 1 and center 2, all patients diagnosed with BVP at the outpatient clinic of the Department of Otorhinolaryngology were asked to participate in the study. These studies consisted of a full day of clinical testing [e.g., caloric test, horizontal and vertical vHIT, torsion swing test, ocular vestibular evoked myogenic potentials (oVEMP), and/or cervical vestibular evoked myogenic potentials (cVEMP)]. In center 3, all patients presented with BVP at the outpatient clinic of the Department of Neurology within the study period were included in the study. Subjects below the age of 18 and subjects who were not able to stop vestibulosuppressive medication were excluded from participation in this study.

### Vestibular Testing

All centers performed vestibular testing to confirm a BVP diagnosis, although the number of tests performed differed between centers. In center 1, vestibular testing included electronystagmography with caloric and rotatory chair testing, as well as horizontal and vertical vHIT and c- and oVEMPs. In center 2, subjects underwent electronystagmography with caloric and rotatory chair testing, horizontal and vertical vHIT, and cVEMPs. In center 3, videonystagmography with caloric testing was performed, together with a horizontal vHIT. An overview of different tests performed in each center is shown in Supplementary Table 1 of the supplementary materials (SM).

#### The Caloric Test

An extensive description of caloric testing was described previously (44). To summarize, in all centers, bithermal caloric testing was performed in both ears whilst patients were in supine position with a forward head inclination of 30°. Each irrigation lasted 30 s with a volume of at least 250 ml of water in centers 1 and 2 and at least 100 ml of water in center 3, for both cold (30°C) and warm (44°C) irrigations with a 5-min stimulus interval between irrigations (Variotherm Plus device, Atmos Medizin Technik GmbH, Lenzkirch, Germany for all three centers). Eye movements were recorded using electronystagmography with self-adhesive electrodes at centers 1 and 2 (Blue sensor, Ambu, Denmark) and with videonystagmography at center 3 (Interacoustics, Munich, Germany). The maximum peak slow phase eye velocity at the culmination phase (°/s) was measured (KingsLab 1.8.1, Maastricht University, Maastricht, The Netherlands at center 1; Nystagliner, Toennies, Germany at center 2; Interacoustics, Munich, Germany at center 3).

#### Torsion Swing Test

During the torsion swing test, patients were seated in a servo-controlled rotatory chair in complete darkness with their eyes open (Ekida GmbH, Buggingen, Germany at center 1 and ServoMed AB, Varberg, Sweden at center 2). Sinusoidal rotatory stimulation was performed at 0.1 Hz at center 1 and 0.05 Hz at center 2 with a peak velocity of 60°/s. Again, eye movements were recorded with electronystagmography with self-adhesive electrodes (Blue sensor, Ambu, Denmark in both center 1 and 2) and the VOR gain was calculated as the ratio between peak eye velocity and peak head velocity (KingsLab 1.8.1, Maastricht University, Maastricht, The Netherlands at center 1; Nystagliner, Toennies, Germany at center 2).

#### Video Head Impulse Test

The horizontal vHIT and the vHIT in the Right-Anterior-Left-Posterior (RALP) and Left-Anterior-Right-Posterior (LARP) canal planes were performed using the Video-Head Impulse Test device from Otometrics at center 1 and 2 (Otometrics, Taastrup, Denmark). At center 3, horizontal vHIT was performed using the Eye-SeeCam (Interacoustics, Munich, Germany). The testing method was described previously (45, 46). In brief, the technician stood behind the subject (who was sitting on a static chair) and held their head firmly without touching the goggles. The subject was instructed to maintain visual fixation on an earth-fixed target at a distance of 2 m at centers 1 and 2 and 1.8 m at center 3. Head impulses comprised fast unpredictable, low-amplitude (±20°) head movements in the horizontal plane (all three centers, peak head velocity > 150°/s) and in the RALP and LARP planes (center 1 and 2, peak head velocity > 100°/s). The Otometrics system defines the VOR gain as the ratio of the area under the eye velocity curve to the area under the head velocity curve from the impulse onset until the head velocity drops to zero again (47). The inter-acoustics system divides the eye and head velocity at a certain point in time (around 60 ms after impulse onset) (46).

#### Vestibular-Evoked Myogenic Potentials

Both centers 1 and 2 used the Neuro-Audio system with electromyographic software (v2010, Neurosoft, Ivanovo, Russia) and self-adhesive electrodes (Blue sensor, Ambu, Denmark) to record the o- and/or c-VEMPS. cVEMPs were measured over the sternocleidomastoid muscle after stimulating the ipsilateral vestibular organ with air-conducted tone bursts of 500 Hz, provided via inserted earphones at a stimulation rate of 13 Hz. oVEMPs were measured over the inferior oblique muscle after stimulating the contralateral vestibular organ with the same stimulation parameters as for cVEMPs. Details on the procedure have been published previously (44, 48, 49). In brief, for cVEMPS, subjects were in a supine position with their back tilted at an angle of 30° from the horizontal plane and were instructed to turn their head away from the stimulus and to lift their head up slightly. A total of 200 EMG traces with a minimum rectified voltage of 65 μv and a maximum rectified voltage of 205 μv were accepted. A visual feedback system (v2010, Neurosoft, Ivanovo, Russia) provided patient feedback to maintain correct muscle contraction. For oVEMPS, subjects were in a supine position and were instructed to keep their gaze fixed on a focus point 30 degrees behind the head to achieve superomedial gaze. A minimum of 300 EMG traces were accepted.

Vestibular-evoked myogenic potentials (VEMPs) were first recorded starting at maximum stimulus intensities of 130 dB sound pressure level (SPL) (center 1) or 95 dB hearing level (HL) (center 2). Then recordings were attempted again using stimulus amplitudes successively decreasing by 5 dB at each step. Thresholds were determined in consensus between two independent technicians at the level where a biphasic wave response was present. When no typical biphasic wave was found at 130 dB SPL at center 1 or 95 dB HL at center 2, a patient was considered to have an absent c- or oVEMP response.

### Data Collection, Processing, and Analysis

The caloric test was performed in all three centers. The torsion swing test was performed in centers 1 and 2, but at different frequencies (0.1 and 0.05 Hz respectively). Since the frequency of sinusoidal rotatory stimulation at center 2 differed from the frequency stated in the BVP diagnostic criteria, the patients from center 2 were not included in this analysis based on their VOR gain measured during the torsion swing test alone. As described above, the horizontal vHIT was performed in all three centers, vertical vHIT and cVEMPs in centers 1 and 2, and oVEMPs only in center 1. Therefore, the amount of data available for analysis differed between tests.

IBM SPSS Statistics version 25 (Armonk, NY: IBM Corp.) and R version 3.5.2 (R Foundation for Statistical Computing, Vienna, Austria) were used for data analysis. Descriptive statistics were used to describe the basic features of the data (e.g., percentages). Non-parametric methods were applied to determine the significant differences between the test results (e.g., Kruskal Wallis H test with *post hoc* Dunn's test and Mann-Whitney *U* test). *P*-values ≤ 0.05 were considered significant and were adjusted and reported with Benjamini-Hochberg correction for multiple testing. Fisher's exact test and the Chi-squared test were used to compare proportions of categorical outcomes.

Before the data was analyzed extensively, it was checked whether the data between the centers could be pooled. Caloric test results differed between the three centers (χ2(2) = 40.8, *p* <0.001), therefore, the data could not be pooled. The torsion swing test results from center 1 and center 2 could not be pooled since the frequency of sinusoidal rotatory stimulation at center 1 (0.1 Hz) differed from center 2 (0.05 Hz) and the results were significantly different (Mann-Whitney U = 1,954.5, *p* = 0.002). No significant differences for vHIT results between centers for five out of six semicircular canals were found (Kruskal-Wallis H test, *p* > 0.05). The left horizontal canal showed a significant difference between centers (χ2(2) = 7.2, *p* = 0.029), however the Levene's test for homogeneity of variance did not show a significant difference (*F* = 1.32, *p* = 0.192). Therefore, the vHIT results of all centers were pooled per canal.

The VEMP results were categorized in absent vs. present responses (i.e., when no typical biphasic wave was found at 130 dB SPL at center 1 or 95 dB HL at center 2, a patient was considered to have an absent c- or oVEMP response). cVEMPs were analyzed for each center separately since 1) the decibel measurement level differed between center 1 (dB SPL) and center 2 (dB HL) and 2) the Chi-squared test showed that there was a significant association between centers and absent vs. present cVEMP responses (χ(2) = 8.57, *p* = 0.014).

To investigate the patterns of vestibular impairment in BVP in general, the vestibular test results were first analyzed using descriptive statistics to describe the basic features of the data. Subsequently, the results were interpreted according to the Bárány diagnostic criteria for BVP, which included a reduced bithermal caloric response (sum of bithermal maximal peak slow phase velocity <6°/s bilaterally) and/or a VOR gain <0.1 during the torsion swing test at 0.1 Hz and/or a bilaterally reduced horizontal vHIT gain of <0.6 (3). To investigate patient eligibility for vestibular implantation regarding the results from vestibular reflex testing, vestibular test results were interpreted according to the vestibular implantation criteria, which included a bilaterally reduced or absent angular VOR function documented by at least one of the major criteria and all minor criteria (i.e., in case only one or two major criteria were met, the remaining tests should comply the minor criteria). The major criteria included a reduced bithermal caloric response (sum of bithermal maximal peak slow phase velocity ≤ 6°/s bilaterally), a reduced horizontal VOR gain ≤ 0.1 during the torsion swing test at 0.1 Hz, and a pathological horizontal VOR gain ≤ 0.6 bilaterally with at least one vertical VOR gain <0.7 bilaterally, measured with vHIT. The minor criteria included a reduced bithermal caloric response (sum of bithermal maximal peak slow phase velocity <10°/s bilaterally), a reduced horizontal VOR gain <0.2 during torsion swing test at 0.1 Hz, and pathological VOR gains of at least two semicircular canals <0.7 bilaterally, measured by vHIT (43).

Hierarchical cluster analysis was applied to explore and visualize patterns of vestibular impairment with respect to etiology. Cluster analysis requires complete cases (i.e., no missing data), therefore, only patients with complete data for caloric testing, torsion swing test, horizontal and vertical vHIT, cVEMPs, and oVEMPS were included (i.e., 45 patients from center 1). Before clustering, the data were standardized in Z-scores (i.e., the individual scores minus the mean, divided by the standard deviation), in order to have the variables weigh equally in the cluster analysis. Ward's method with the distance measure squared Euclidian distance was used since Ward's method has the highest agglomerative coefficient compared with the other hierarchical clustering methods. The silhouette method was used to determine the optimum number of clusters (50). Hierarchical cluster analysis resulted in two dendrograms with etiology on the x-axis and vestibular tests results on the y-axis. A heatmap was created. Each column represented one subject and each row represented the output of a specific vestibular test. A “relatively bad (vestibular) score” was illustrated by lower Z scores in the color red. A “relatively good (vestibular) score” was illustrated by higher Z scores in the color blue. After performing the analysis, etiology and patient characteristics, and vestibular test results were compared between clusters.

### Ethical Considerations

The study was approved by the local ethical committee of center 1 (protocol number NL52768.068.15 / METC 151027), the local ethical committee of center 2 (protocol number 16/42/426), and the local ethical committee of center 3 (project number 20-174). The study was registered on trialregister.nl [center 1, Trial NL5446 (NTR5573)]and ClinicalTrials.gov [center 2, (NCT03690817)]. All study participants gave their written informed consent prior to inclusion in the study.

## Results

### Patient Characteristics

**Figure 1:**
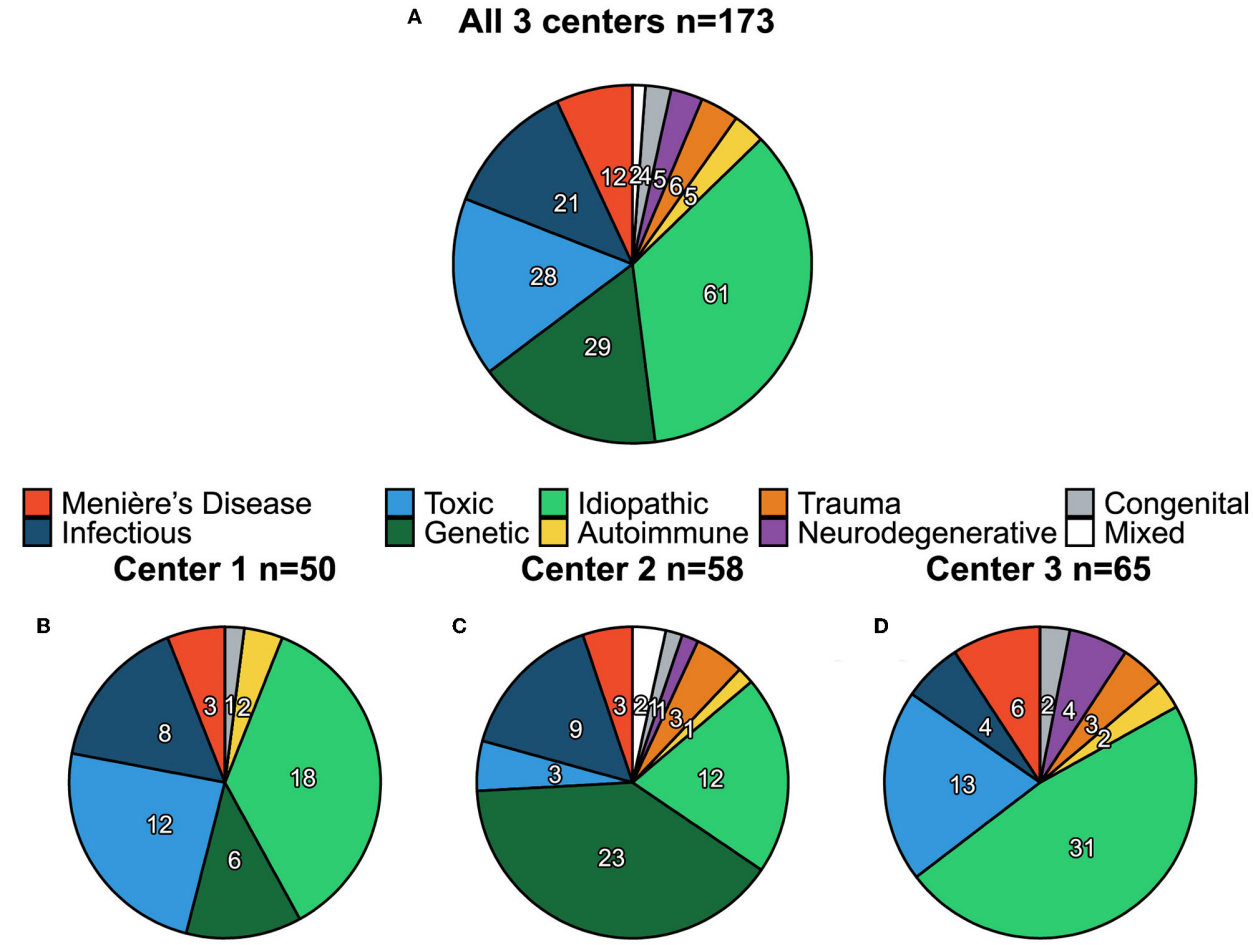
Distribution of etiology of bilateral vestibulopathy (BVP) for all three centers combined **(A)** and per center separately **(B–D)**. Numbers shown in each pie chart represent the count (*n*) of each etiology.

A total of 173 patients (50 from center 1, 58 from center 2, and 65 from center 3, 53% males) were included in this study with a mean age of 60 ± 15 years (range 19–91 years). A diagnosis of the underlying etiology of BVP could be identified in 112 out of the 173 patients. Genetic disorders (*n* = 29, 17%), ototoxicity (*n* = 28, 16%) and infectious disorders (*n* = 21, 12%) were the most common etiologies. Less frequently, the cause of BVP was due to Menière's Disease (*n* = 12, 7%), (head)trauma (*n* = 6, 4%), auto-immune disease (*n* = 5, 3%), neurodegenerative disorders (*n* = 5, 3%), or congenital disorders (*n* = 4, 2%). Two patients presented with a mixed etiology (vestibular schwannoma on one side and idiopathic etiology on the other side). In approximately one-third of the cases (*n* = 61, 35%), no underlying etiology could be identified. The distribution of etiology (Figure 1) was significantly different between centers (Fisher's Exact Test *p* < 0.01). A detailed overview of all etiologies is shown in Supplementary Table 2.

### Vestibular Function

#### Vestibular Test Results

**Figure 2:**
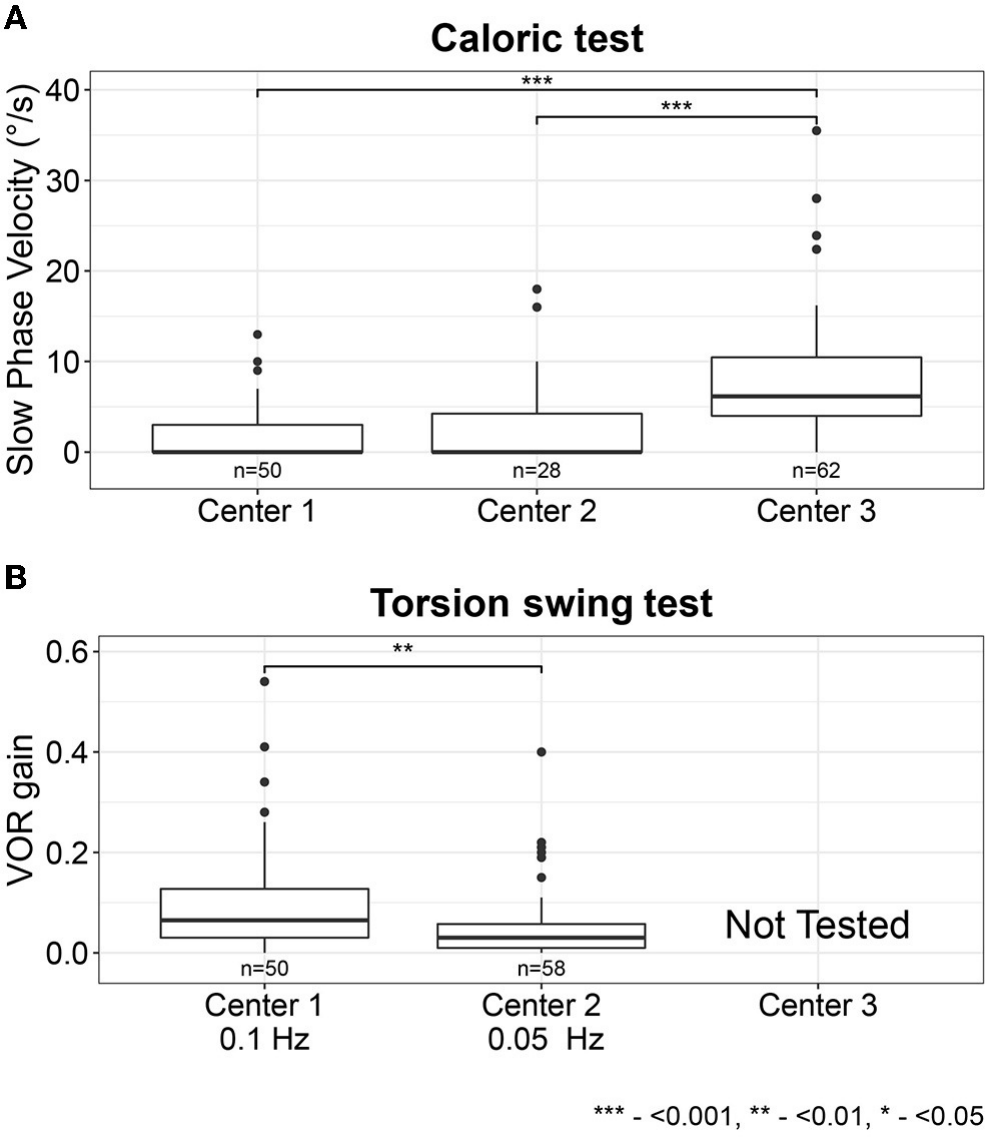
Vestibular test results for the caloric test (sum of the bithermal maximal peak slow phase velocity bilaterally) **(A)** and torsion swing test (VOR gain) **(B)** per center. Each box plot represents the 25 to 75 percentiles, bold black lines the median, dots the outliers, and asterisks (*) illustrate statistically significant differences. Statistical significance levels: ***p < 0.001, **p < 0.01, *p < 0.05.

Median caloric test results were significantly higher for center 3 (6.2°/s) compared with centers 1 and 2 (both 0°/s) (χ2(2) = 39.6, *p* < 0.001, Figure 2). No significant differences were found between the median caloric test results for centers 1 and 2. The torsion swing test results from center 1 (0.1Hz) were significantly higher compared with center 2 (0.05 Hz) (Mann-Whitney *U* = 1,954.5, *p* = 0.002, Figure 2).

**Figure 3:**
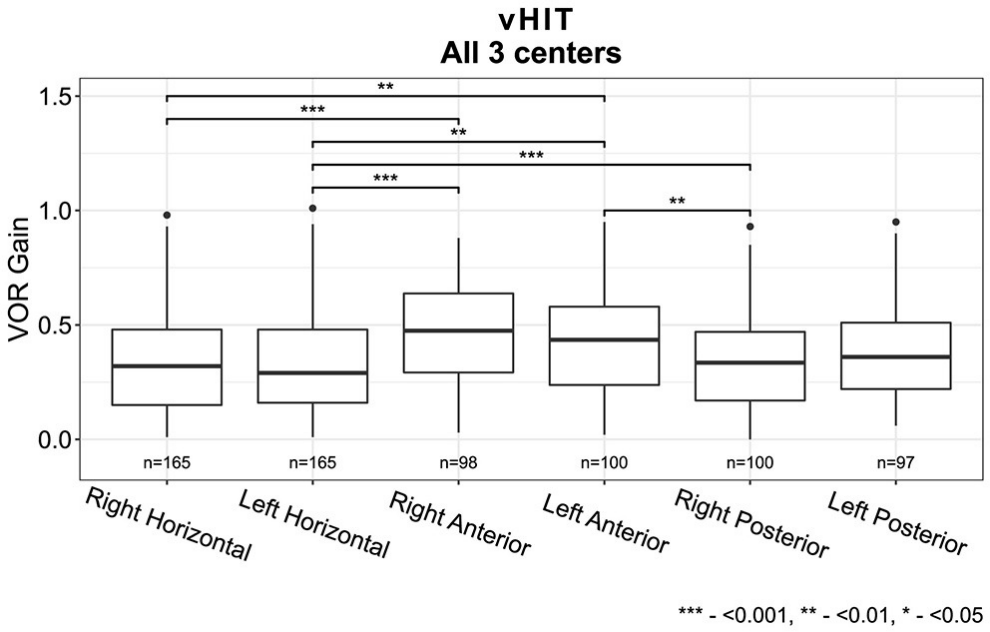
Vestibulo-ocular reflex (VOR) gain for all six semi-circular canals measured with video Head Impulse Test for all three centers combined (Horizontal canals: center 1, 2 and 3, Vertical canals: center 1 and 2). Each box plot represents the 25 to 75 percentiles, bold black lines the median, dots the outliers, and asterisks (*) illustrate statistically significant differences.

The vHIT results showed a median VOR gain below 0.5 for all semicircular canals, with the lowest VOR gain measured at the horizontal canals and the highest VOR gain measured at the anterior canals (χ2(5) = 35.5, *p* <0.001, Figure 3). After analyzing the data separately per center, this trend was detectable in both centers 1 and 2 but only significant in center 2 after correction for multiple comparisons (χ2(5) = 35.7, *p* < 0.001, Supplementary Figures 1, 2 and Supplementary Table 3).

**Figure 4:**
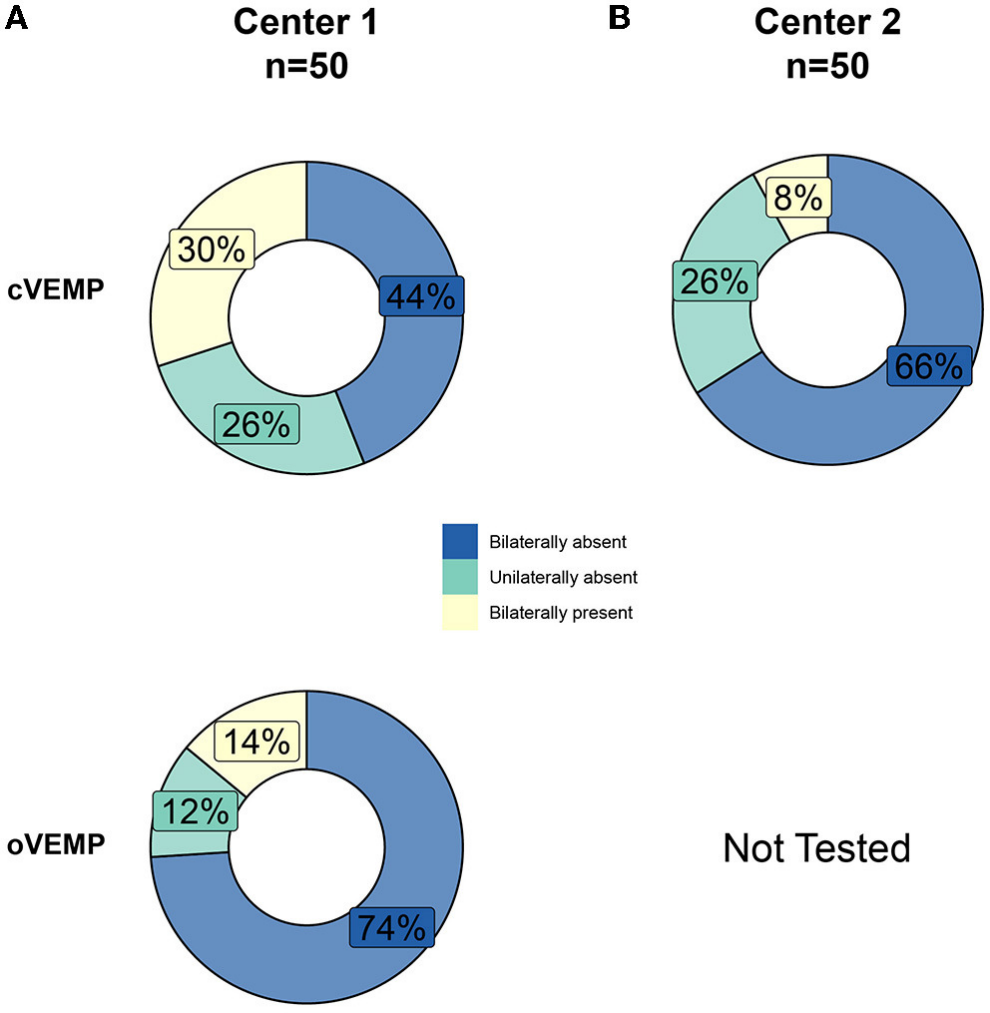
Percentages of bilaterally absent, unilaterally absent, and bilaterally present cervical and ocular vestibular evoked myogenic potentials (cVEMPs, oVEMPs) of patients with BVP at center 1 **(A)** and center 2 **(B)**.

The percentage of bilaterally absent cVEMP responses was higher in center 2 compared with center 1 (66 and 44% respectively, χ(2) = 8.57, *p* = 0.014). When looking at the cVEMP and oVEMP responses at center 1, the rates of bilaterally absent oVEMP responses were higher compared to bilaterally absent cVEMP responses (74 vs. 44% respectively, χ(2) = 9.30, *p* = 0.010) (Figure 4).

#### Vestibular Impairment According to the Bárány Diagnostic Criteria for BVP

**Figure 5:**
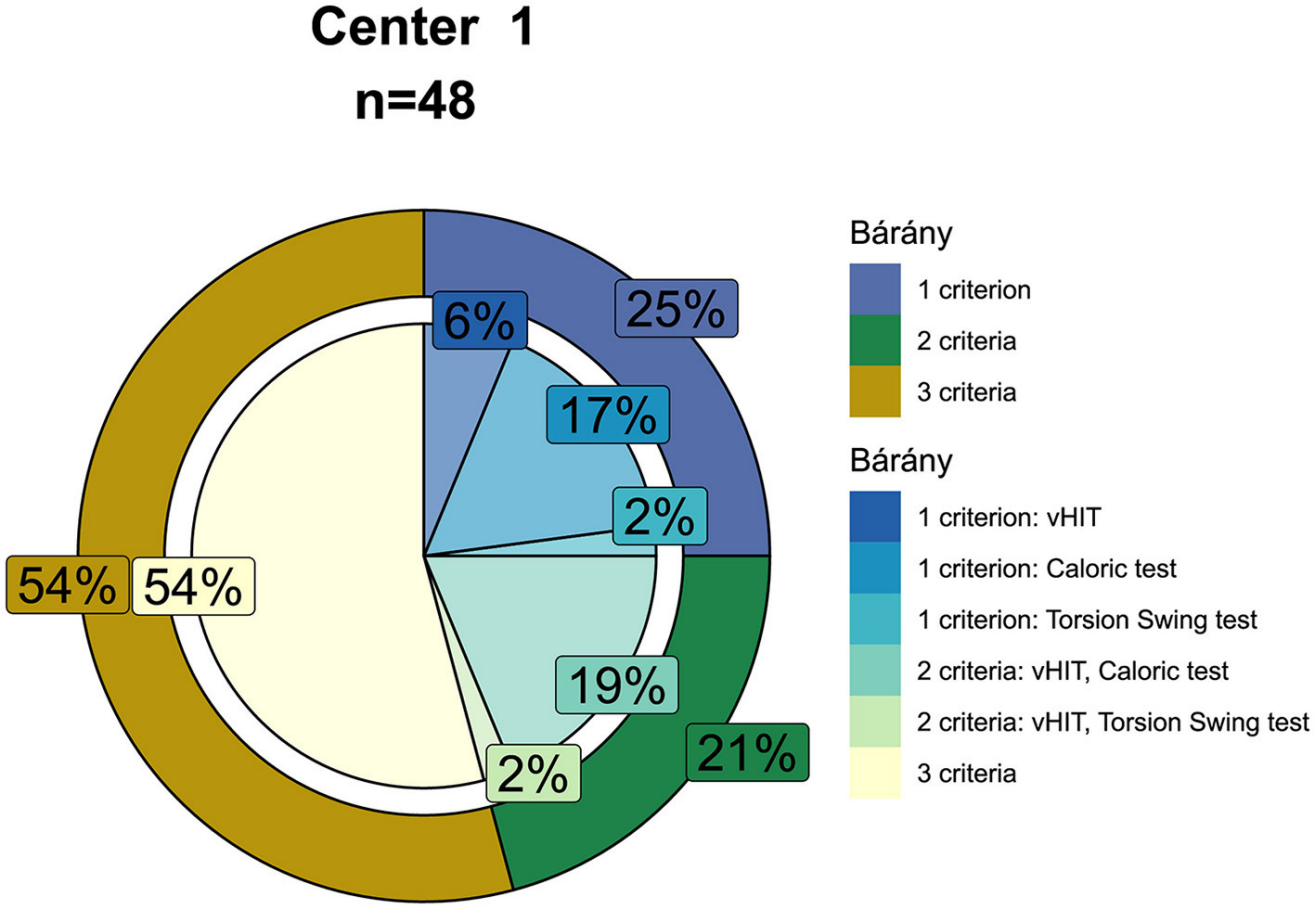
Percentages of patients with BVP meeting one, two or three of the diagnostic criteria of the Bárány Society (shown in the outer circle, i.e., a reduced bithermal caloric response with a sum of the bithermal maximal peak slow phase velocity <6°/s bilaterally and/or a VOR gain <0.1 during torsion swing test at 0.01 Hz and/or a bilaterally reduced horizontal video Head Impulse Test gain of <0.6). The inner-circle shows the percentages of which tests are met by patients meeting one or two of the diagnostic criteria. Only cases without missing data for caloric testing, torsion swing test, and horizontal video Head Impulse Test were included (center 1, *n* = 48).

Regarding the cases without missing data for caloric testing, torsion swing test, and horizontal vHIT, the majority of the patients (54%) met three of the criteria of the Bárány Society described earlier, whereas 21% met two of the Bárány criteria, and 25% only met one criterion. In the group of patients who met two out of three Bárány criteria, an impaired VOR gain measured with vHIT combined with a reduced caloric response was most prevalent (19%). In the group of patients who only met one of the Bárány criteria, a reduced caloric response was most prevalent (17%), followed by an impaired VOR gain measured with vHIT (6%) and torsion swing test (2%) (Figure 5).

**Figure 6:**
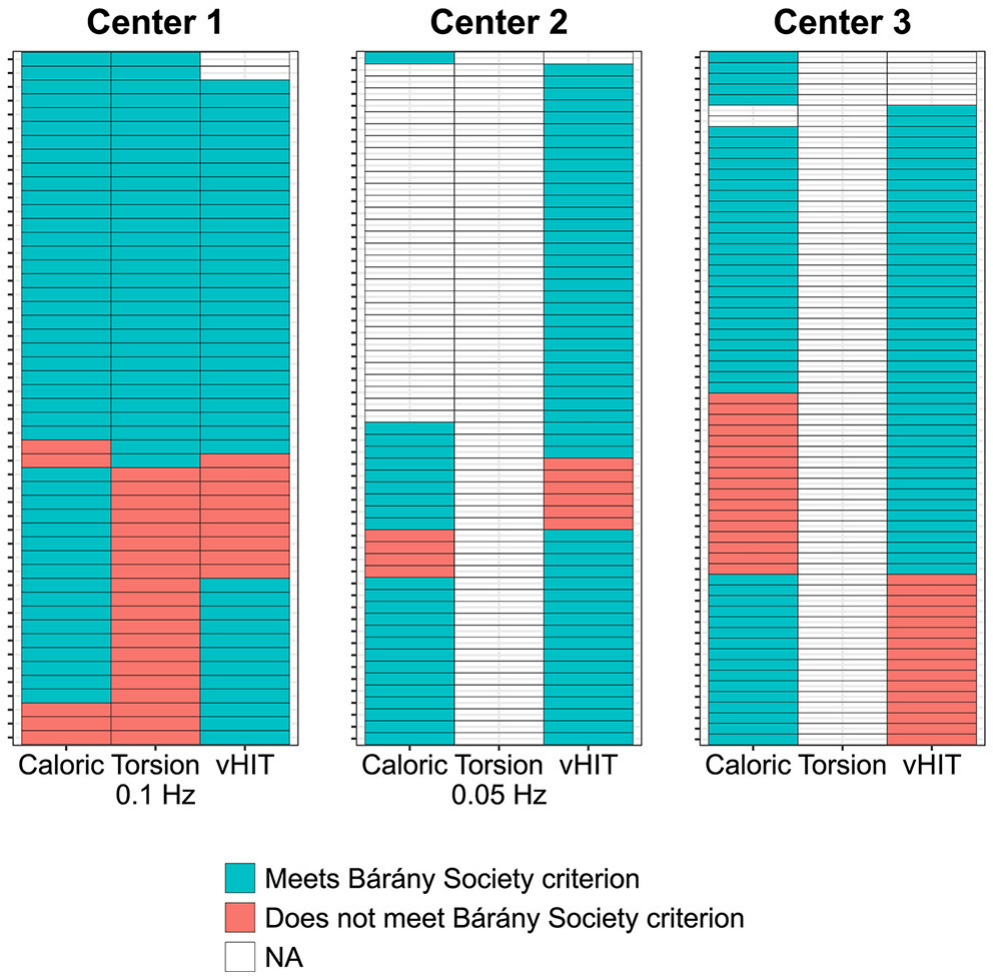
Distribution of patients with BVP meeting the diagnostic criteria of the Bárány Society, presented for each test separately in the color blue (i.e., for the caloric test a reduced response with a sum of bithermal maximal peak slow phase velocity <6°/s bilaterally; for the torsion swing test (0.1 Hz) an impaired VOR gain <0.1; for horizontal video Head Impulse Test (vHIT) a bilaterally reduced VOR gain <0.6). Patients not meeting the diagnostic criteria for each test separately are indicated with the color red. Each column represents one of the diagnostic criteria per center; each row represents one subject per center. NA, no data available.

When considering the total study population, the caloric test and horizontal vHIT more often indicated horizontal semicircular canal impairment than the torsion swing test (Figure 6). For example, in center 1 only one patient was diagnosed with BVP according to the Bárány criteria based on the torsion swing test alone, whereas the rest of the population was diagnosed with the caloric test or horizontal vHIT or both.

#### Patient Eligibility for Vestibular Implantation According to the Implantation Criteria

**Figure 7:**
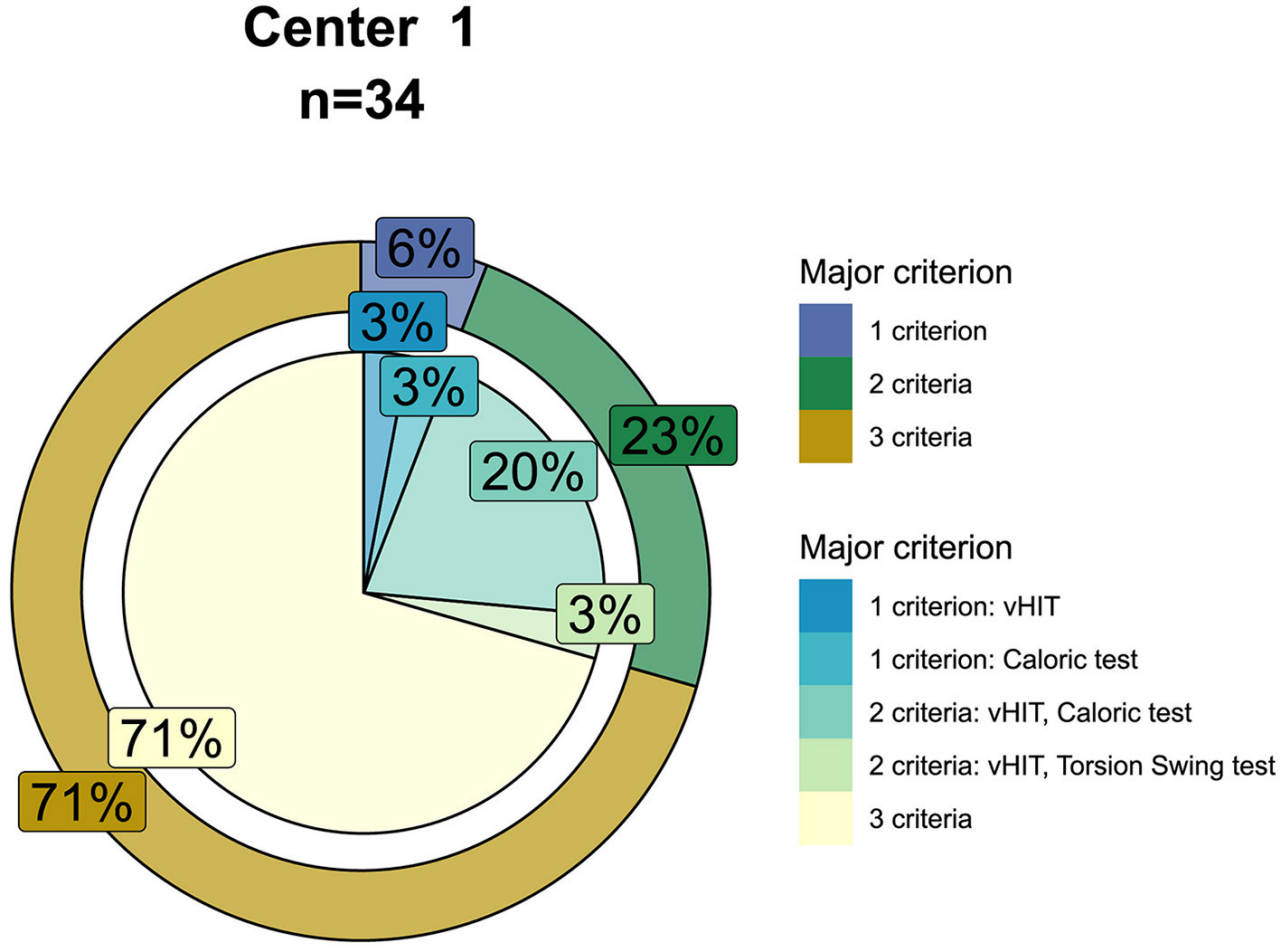
Seventy-six percent of the patients met the criteria for vestibular implantation (*n* = 34). For this group, the outer circle shows the percentages of patients meeting one, two, or three of the major implantation criteria (i.e., for caloric testing bilateral impaired caloric responses with a sum of bithermal maximal peak slow phase velocity ≤ 6°/s, for torsion swing test a reduced VOR gain ≤ 0.1 and for vHIT a bilaterally reduced horizontal VOR gain ≤ 0.6 combined with at least bilaterally one vertical VOR gain <0.7). The inner-circle shows the percentages of which tests are met by patients meeting one or two of the major implantation criteria.

Regarding the cases without missing data for caloric testing, torsion swing test, and horizontal and vertical vHIT (*n* = 45), the majority of the patients (*n* = 34, 76%) met the implantation criteria. A total of 71% of this group met three of the major criteria, whereas 24% met two major criteria, and 6% only met one major criterion. In the group of patients who met two out of three major implantation criteria, an impaired VOR gain measured with vHIT combined with a reduced caloric response was most prevalent. In the group of patients who only met one of the major implantation criteria, a reduced caloric response and an impaired VOR gain measured with vHIT were equally common. None of the patients only met the major implantation criteria for the torsion swing test (Figure 7).

### Vestibular Function and Possible Relations to Underlying BVP Etiology

**Figure 8:**
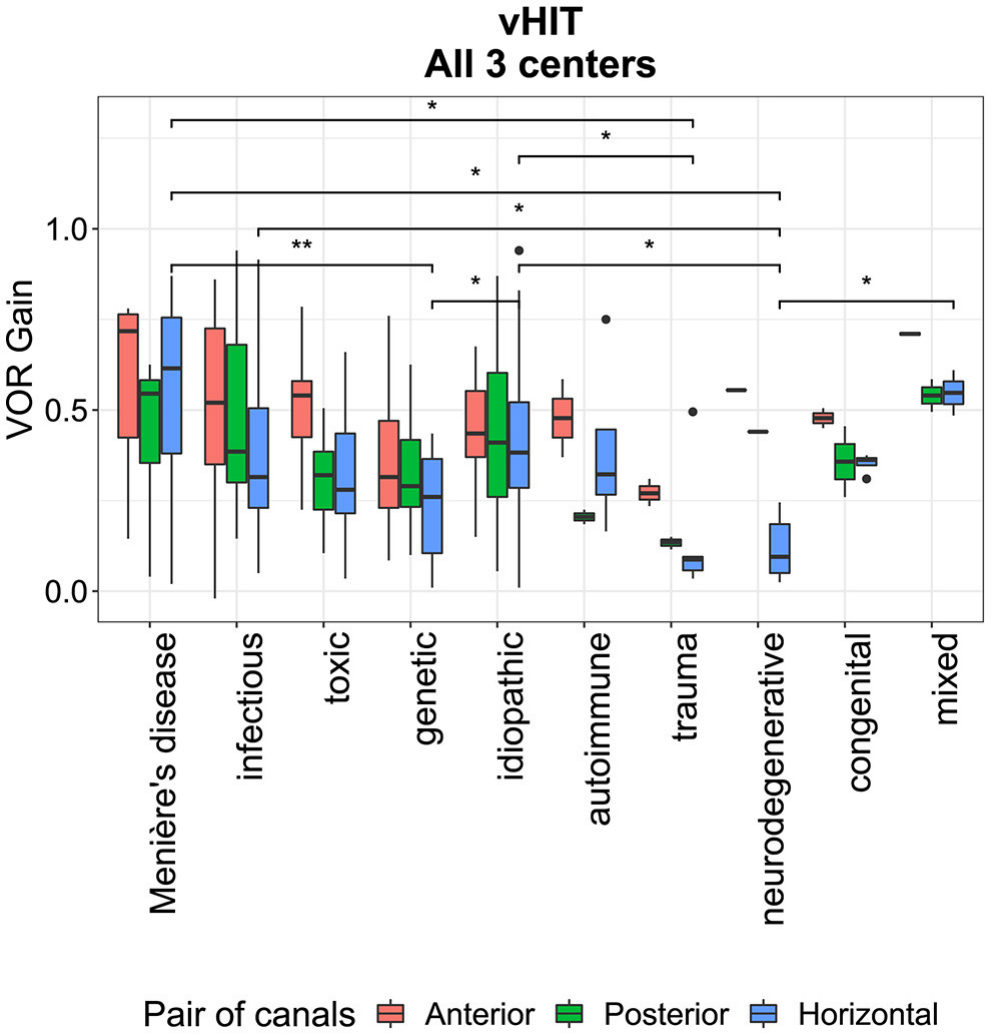
Vestibular Ocular Reflex (VOR) gain per etiology for all three pairs of semi-circular canals (i.e., horizontal, anterior, and posterior canals) measured with video Head Impulse Test, presented for all three centers combined. Each box plot represents the 25–75 percentiles, bold black lines the median, dots the outliers and asterisks (*) illustrate statistically significant differences.

The median vestibular test results for caloric testing and torsion swing test did not differ between different etiologies (Kruskal-Wallis H test with *post hoc* Dunn test and Benjamini Hochberg correction *p* > 0.05, Supplementary Tables 4, 5). The vHIT results did not differ between etiologies for the anterior and posterior canals in the total group (Kruskal Wallis H test, *p* > 0.05, Supplementary Table 4). However, the horizontal vHIT results were significantly lower in the total group for neurodegenerative disorders compared with the idiopathic group, infectious disorders, Menière's Disease, and the mixed etiology group. The horizontal vHIT results were also significantly lower for genetic disorders compared with the idiopathic group and Menière's Disease. Lastly, horizontal vHIT results were significantly lower for (head)trauma compared with the idiopathic group, Menière's Disease and mixed etiology (Kruskal-Wallis H test with *post hoc* Dunn test and Benjamini Hochberg correction *p* < 0.05, Figure 8 and Supplementary Tables 4, 5). After analyzing the data separately per center, some trends were detectable per center (e.g., lower horizontal vHIT results for genetic disorders in center 1 and lower horizontal vHIT results for (head)trauma in center 2), however, no significant differences were found except for lower horizontal vHIT results for neurodegenerative disorders compared with the idiopathic group and Menière's Disease in center 3 (Kruskal-Wallis H test with *post hoc* Dunn test and Benjamini Hochberg correction, Supplementary Table 5, Supplementary Figure 3).

Regarding VEMPs, the highest fraction (≥50%) of bilaterally absent cVEMP responses in center 1 was found in patients with ototoxic, infectious, autoimmune, and congenital etiologies, whereas in center 2 almost all etiologies showed high fractions (>60%) of bilaterally absent cVEMP responses (except neurodegenerative disorders). Next to this, all etiologies showed high fractions (≥50%) of bilaterally absent oVEMP responses (center 1) (Supplementary Figures 4, 5). No significant differences were found between the different etiologies and the proportion of patients with pathologic VEMP responses (Fisher's exact test *p* =0.52 and *p* = 0.99 for cVEMPs centers 1 and 2 respectively and Fisher's exact test, *p* = 0.36 for oVEMPs center 1).

**Figure 9:**
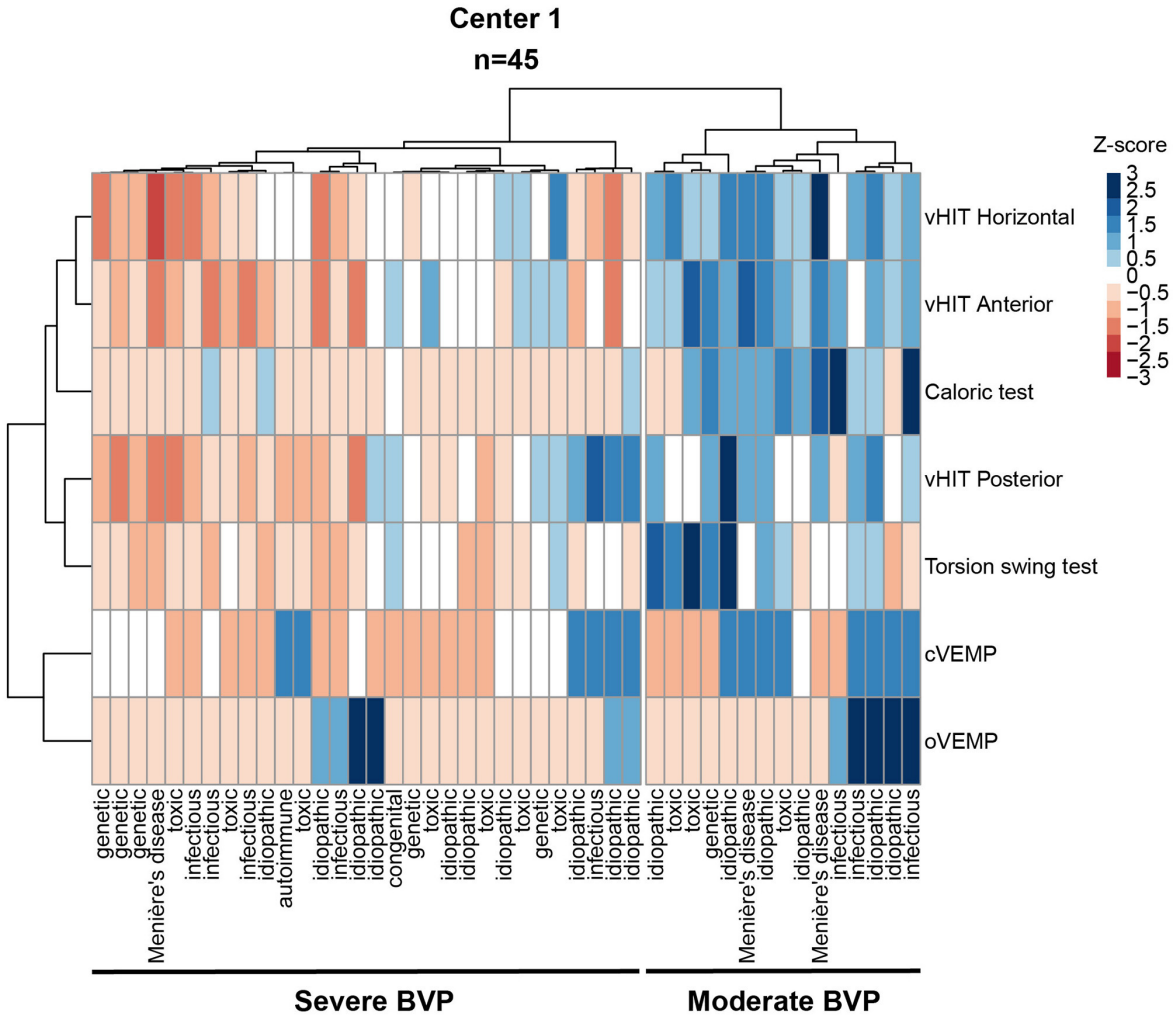
Heatmap as a result of hierarchical cluster analysis with two dendrograms; Each column represents one subject; each row represents the results of a specific vestibular test. A “bad (vestibular) score” (i.e., low scores on tests of vestibular reflexes) is illustrated by lower Z scores in the color red. A “relatively good (vestibular) score” (i.e., relative high scores on tests of vestibular reflexes) is illustrated by higher Z scores in the color blue. Bold underlining indicates the 2 clusters; “Cluster 1, severe BVP” and “Cluster 2, moderate BVP”. Only cases without missing data for caloric testing, torsion swing test, and horizontal and vertical vHIT, and o- and c-VEMPs (*n* = 45). vHIT, video Head Impulse Test; oVEMP, ocular Vestibular Evoked Myogenic Potential; cVEMP, cervical Vestibular Evoked Myogenic Potentials.

To investigate the pattern of vestibular impairment and its relation with etiology, hierarchical cluster analysis was performed, which resulted according to the silhouette method in two clusters (Figure 9). The first cluster “severe BVP” (*n* = 30; 47% female; mean age 58 years) showed overall lower median vestibular test results compared with the second cluster “moderate BVP” (*n* = 15; 60% female; mean age 60 years), which is illustrated by lower Z scores in the color red for relatively low vestibular scores and in the color blue for relatively high vestibular scores compared with the study group in Figure 9. This was significant for the caloric test, torsion swing test, horizontal vHIT, vertical vHIT (Mann-Whitney *U, p* <0.001), and cVEMP (Fisher's Exact Test *p* = 0.04). A detailed overview of all median test results and statistics can be found in Supplementary Tables 6, 7. Next to this, the distribution of the amount of Bárány criteria met, was significantly different among clusters: cluster 1 “severe BVP” consisted of patients who predominantly met three criteria, whereas cluster 2 “moderate BVP” mainly included patients who only met one criterion (Supplementary Figure 6).

Some etiologies were more prevalent in one of the two clusters. For example, genetic disorders were more prevalent in the first cluster “severe BVP”, whereas Menière's Disease was more prevalent in the second cluster “moderate BVP” (Supplementary Figure 7). However, no significant association between etiology and clusters was found (Fisher's Exact Test *p* = 0.854, Supplementary Table 8).

Next to this, some similarities in vestibular reflex tests were found in the cluster analysis (Figure 9, left dendrogram). It was observed that the horizontal and anterior vHITs were arranged close to each other and to caloric testing; the posterior vHIT was located close to the torsion swing test; and oVEMPs and cVEMPs formed a pair.

## Discussion

This study provided a description of patterns of vestibular impairment and its relation to BVP etiology in a cohort of 173 patients with BVP from 3 centers, diagnosed according to the Bárány Society criteria. Vestibular function was measured using the caloric test, torsion swing test, horizontal and vertical vHIT, cVEMPs, and/or oVEMPs. Etiologies were split into 10 separate groups (i.e., idiopathic, genetic disorders, ototoxicity, infectious disorders, Menière's Disease, (head)trauma, auto-immune disease, neurodegenerative disorders, congenital disorders, and mixed etiology). The patterns of the vestibular impairment and their relation to BVP etiology are discussed below.

### Patterns of Vestibular Impairment

Overall, this study demonstrated that more than half of patients diagnosed with BVP according to the Bárány Society diagnostic criteria met all three criteria regarding vestibular testing. In patients who only met one or two of the criteria, the caloric test and horizontal vHIT criteria were most often met, in contrast to the torsion swing test criterion. The same trend was found when adhering to the vestibular implantation criteria. However, since the implantation criteria also include the vertical semicircular canals, the percentage of patients meeting the horizontal and vertical vHIT implantation criterion was lower compared with the percentage of patients meeting the horizontal vHIT diagnostic (Bárány Society) criterion. Despite the fact that the Bárány Society diagnostic criteria and vestibular implantation criteria seem to be, to some extent, similar to each other, they set different goals and have therefore several substantial differences. As stated above, the vestibular implantation criteria include all three semicircular canals. As a consequence, the vertical canal function should be considered next to the horizontal canal function. Additionally, although the major and especially the minor implantation criteria are less strict in terms of cut-off values for the caloric test, torsion swing test, and horizontal vHIT compared with the Bárány Society diagnostic criteria, a potential implant candidate must meet all the implantation criteria (Figure 5 vs. Figure 7). Therefore, 76% of the patients diagnosed with BVP according to the Bárány Society diagnostic criteria were eligible for implantation according to the vestibular implantation criteria. Furthermore, apart from vestibular reflex testing, the vestibular implantation criteria also include assessment of comorbidities and eligibility to undergo surgery (43). Therefore, only a subgroup of the BVP population will be eligible for implantation.

When investigating vestibular test results per test and per center separately, it was observed that the slow phase eye velocities measured during the caloric test were significantly higher in center 3 compared with centers 1 and 2 (Figure 2). This can be explained by different factors, varying from differences in caloric testing methods used (namely electronystagmography at center 1 and 2 and videonystagmography at center 3) which can result in different phase velocities values due to different blink detection and image processing algorithms used (51), to different patient populations included in each center (2). Next to this, torsion swing test results were significantly higher in center 1 compared with center 2, which can be explained by the differences in the used frequency (0.1 Hz at center 1 and 0.05 Hz at center 2): the vestibular system is more sensitive for rotations at 0.1 Hz than 0.05 Hz, leading to a higher response (i.e., VOR gain) (2, 52, 53). This sensitivity might also account for the fact that the diagnostic torsion swing test criterion was less often met than the criteria of the caloric test. After all, since the vestibular system has its optimum sensitivity around the frequencies tested by the torsion swing test at 0.1 Hz (in contrast to the frequencies tested by the caloric test), a uniform decrease in semicircular canal function across all frequencies might result in losing responses to caloric testing first. Although frequencies tested with vHIT are also within the optimum frequency range of the vestibular system, vHIT more often indicated horizontal semicircular canal impairment than the torsion swing test. Therefore, it might be hypothesized that the vestibular system shows an impairment for conditions earlier that demand a relatively large vestibular output in response to high accelerations and velocities. This hypothesis needs further investigation. Nevertheless, the results of this study showed that the response to torsion swing testing might be preserved the longest (53). Therefore, the torsion swing test is least sensitive in detecting BVP, but most sensitive in measuring residual vestibular function, whereas caloric testing and vHIT seem to be more sensitive for measuring vestibular impairment (2).

The variability of VEMP responses in this study is in line with results from previous studies, which also demonstrated the wide range of otolith function (as measured with c- and oVEMPs) in patients with BVP (36, 38, 54). This variability could be explained by the large range of VEMP responses present in normal subjects, the heterogeneous nature of BVP, and the nature of VEMP testing itself (e.g., it is still unknown how much residual otolith function needs to be present to produce a synchronous motor discharge) (54). Furthermore, because of the diagnostic inclusion criteria, all of the included patients have horizontal semicircular canal impairment, whereas the function of the other vestibular end organs can have variable degrees of (dys)function. Since the utricle (tested with oVEMPs) projects into the superior branch of the vestibular nerve together with the horizontal semicircular canal, it can be hypothesized that patients included based on horizontal canal impairment also show bilaterally absent oVEMP responses (55). This might explain why rates of bilaterally absent oVEMP responses were higher compared to bilaterally absent cVEMP responses in center 1 (Figure 4) since there is possibly an intact inferior vestibular nerve function on which the saccule projects. Currently, it is not known whether isolated bilateral dysfunction of both otolith organs also causes significant disability (54). Therefore, all vestibular end organs should be evaluated before and after vestibular implantation in order to create awareness about potential damage to intact vestibular structures.

### Contribution of Etiology to Vestibular Impairment

The distribution of etiologies (Figure 1) was significantly different among the three centers, indicating the inhomogeneity of the data. This can potentially be caused by differences in clinical settings, namely ENT clinics (center 1 and center 2) compared with a neurological clinic (center 3). This fact can explain the trend that among all 3 centers the biggest fraction of neurodegenerative and idiopathic patients was observed in center 3, whereas the biggest fraction of infectious and genetic disorders were observed in centers 1 and 2.

The distribution of the vHIT VOR gains between different etiologies indicated several trends, although not every trend proved to be statistically significant (Figure 9). Overall, the vHIT results showed significantly better gains for both anterior canals compared with the horizontal and posterior canals, which corresponds with previous literature (24). The vHIT results did not differ significantly between etiologies for anterior and posterior canals although trends of anterior canal sparing were observed for Menière's Disease, infectious disorders, ototoxicity, trauma, and idiopathic BVP. This is congruent with previous literature (24). Next to this, horizontal vHIT results were significantly lower for neurodegenerative disorders, genetic disorders, and trauma, whereas horizontal vHIT results were significantly higher for Menière's disease and infectious disorders.

Cluster analysis identified two separate clusters of patients with BVP in center 1 (which was the center with the most available vestibular test data) with significant differences in residual vestibular function according to vestibular testing. This was also reflected by the amount of diagnostic and vestibular implantation criteria met between clusters. Cluster 1 “severe BVP” consisted of patients who predominantly met 3 criteria, whereas cluster 2 “moderate BVP” mainly included patients who met only 1 criterion (Supplementary Figure 6). Although, no statistically significant differences were found in the etiology distribution between clusters (Figure 9), a slightly higher prevalence of Menière's Disease was observed in cluster 2 “moderate BVP” that performed “better” in all vestibular tests, whereas the idiopathic and ototoxicity etiologies prevailed in cluster 1 “severe BVP” and performed “worse” (Supplementary Figure 4). This could imply that the contribution of etiology to specific patterns of vestibular impairment might be limited and would eventually result in an overall better or worse vestibular function. Despite some patterns being found for a few BVP etiologies, one should consider every case individually and investigate every part of the vestibular system separately to obtain a full understanding of the vestibular impairment.

### Order of Vestibular Test Outcomes According to Cluster Analysis

The cluster analysis showed similarities in the vestibular reflex tests used in center 1 (Figure 9, left dendrogram). For example, horizontal and anterior vHITs were arranged close to each other and to caloric testing; posterior vHIT was located close to torsion swing test; and oVEMPs and cVEMPs formed a pair. It is quite intuitive for VEMP results to be correlated to each other since the two otolith organs are located next to each other. However, the opposite was found when testing the semicircular canal function. The caloric test, torsion swing test, and horizontal vHIT are aimed to measure horizontal canal function and it could be hypothesized that they would closely correlate to each other. However, this was not observed in the cluster analysis, which showed the close correlation of the anterior and horizontal vHIT results together with the caloric test, and the close correlation of posterior vHIT results with the torsion swing test. The proximity of the horizontal and anterior vHIT in the cluster analysis can be partly explained in terms of anatomy. The horizontal and anterior canals ampullae are located close to each other and project into the same superior vestibular nerve division, whereas the inferior vestibular nerve division receives input from the posterior canals (56). Next to this, as stated before, vHIT and the caloric test seem to be able to indicate vestibular impairment, whereas the torsion swing test is more sensitive to measure the residual vestibular function (53). This could explain why the torsion swing test is not in close proximity to the horizontal vHIT and caloric test in the cluster analysis. The trends found in this cluster analysis differed from the trends described by a previous study (40). For example, differences in the arrangement of the variables after clustering [e.g., horizontal and posterior canals in close proximity to the utriculus according to the previous study (40)]. Furthermore, in contrast to the study presented here, no differences in vestibular impairment were found. This could be the result of different approaches used, namely: (1) Normalization of data using single test results across patient groups (this study) compared with using all vestibular test results within one single patient (40); (2) scoring of vestibular function using results from separate vestibular reflex tests (this study) compared with considering separate vestibular organs [canals and otoliths, (40)]. To sum up, the different study goals could result in a different distribution of patients among clusters and different interpretations regarding etiology, despite the implementation of the same analysis. Therefore, it is advised to investigate all vestibular end organs separately to appreciate the vestibular impairment as a whole.

### Limitations

Most importantly, some etiology groups included small amounts of patients (*n* < 5), which complicated statistical analysis. Next to this, the vestibular implantation criteria include a number of items that are not related to the vestibular reflex testing, which were not considered in this study (e.g., psychological or psychiatric disorders or an ability to undergo surgery). This could imply that the number of patients eligible for implantation in this study is an overestimation. Due to the study setting in centers 1 and 2, it cannot be ruled out that this study potentially suffered from selection bias due to the inclusion of a relative “healthy” BVP population (i.e., patients with severe symptoms would potentially not want to participate in a full day of clinical testing). However, when comparing centers 1 and 2 with center 3, the results indicate that this might not be the case. A potential risk of referral bias in center 1 could not be excluded, since center 1 is involved in research regarding future vestibular implant therapy (43). This could lead to specific referrals or third opinion consultations in this center. Finally, the torsion swing test phase, being the 4th criterion according to Bárány criteria, was not used in this study, because either it was not measured or the automatic calculation algorithm was not considered reliable.

### Future Perspectives

In order to gather a much bigger dataset from different sources that can be pooled and analyzed together, an international standardized approach for vestibular testing will be crucial (57). In particular, (1) different VEMP devices should be compared to each other in order to obtain the relation between stimuli and the threshold values; (2) the same torsion swing frequency and velocity should be used; and (3) raw traces of eye movements in both vHIT [obtained from different devices (46)] and caloric testing (electronystagmography vs. videonystagmography) should be analyzed, since different processing algorithms may lead to a significant difference in results of gain and SPV. In the case of a larger dataset, etiologies can be defined more specifically and at a more pathophysiological and morphological level (e.g., etiologies that lead to fibrosis). Next to this, future research between objective vestibular reflex test results and self-reported symptom severity could provide more insight into the effect of different patterns of vestibular impairment and degree of specific BVP symptoms (e.g., anterior canal sparing and self-reported oscillopsia severity).

## Conclusion

This study provided a description of vestibular function in a large cohort of patients with BVP diagnosed according to the Bárány Society criteria. Overall, this study showed differences in the degree of vestibular impairment measured with different vestibular tests such as caloric test, vHIT, torsion swing test, and VEMPs. More specifically, some tests (i.e., caloric testing and horizontal vHIT) seem to be more sensitive for detecting vestibular impairment, whereas other tests (e.g., torsion swing test) are more suited for measuring residual vestibular function. In addition, no striking patterns of vestibular impairment in relation to etiology were found. Nevertheless, when comparing the Bárány Society diagnostic and vestibular implantation criteria, it was shown that although the implantation criteria are more strict, still 76% of the patients with BVP were eligible for implantation based on vestibular test criteria. It is advised, especially in the research setting, to carefully examine every patient for their overall pattern of vestibular impairment (i.e., all five vestibular end organs), in order to make well-informed and personalized therapeutic decisions.

## Data Availability Statement

The raw data supporting the conclusions of this article will be made available by the authors, without undue reservation.

## Ethics Statement

The studies involving human participants were reviewed and approved by the Medical Ethics Committee of Maastricht, Antwerp and Munich. The patients/participants provided their written informed consent to participate in this study.

## Author Contributions

MP and LS conducted the analysis and wrote the manuscript. FL, BD, and VM ensured data acquisition. RB supervised the writing and edited the manuscript. MS, NG, AP, and JW reviewed the manuscript. All authors contributed to the article and approved the submitted version.

## Funding

LS and FL were supported through funding of MED-EL (Innsbruck, Austria). RB, AP, and NG received funding for travel from MED-EL. MP was supported by the Tomsk State University Development Program (≪Priority-2030≫). MS receives support for clinical studies from Decibel, U.S.A., Cure within Reach, U.S.A. and Heel, Germany. MS distributes M-glasses and Positional vertigo App. The funders had no role in study design, data collection, data analysis, interpretation of data, decision to publish, or preparation of the manuscript.

## Conflict of Interest

MS acts as a consultant for Abbott, AurisMedical, Heel, IntraBio, and Sensorion. MS has received speaker's honoraria from Abbott, Auris Medical, Biogen, Eisai, Grünenthal, GSK, Henning Pharma, Interacoustics, J & J, MSD, NeuroUpdate, Otometrics, Pierre-Fabre, TEVA, UCB, and Viatris. The remaining authors declare that the research was conducted in the absence of any commercial or financial relationships that could be construed as a potential conflict of interest.

## Publisher's Note

All claims expressed in this article are solely those of the authors and do not necessarily represent those of their affiliated organizations, or those of the publisher, the editors and the reviewers. Any product that may be evaluated in this article, or claim that may be made by its manufacturer, is not guaranteed or endorsed by the publisher.
