# Supplementary material for: Patterns of Vestibular Impairment in Bilateral Vestibulopathy and Its Relation to Etiology
Source: Front Neurol. 2022 Mar 21;13:856472. doi: 10.3389/fneur.2022.856472 (PMC8979031; doi:10.3389/fneur.2022.856472)
Supplement: Supplementary file 1 [file Data_Sheet_1.PDF]

Supplementary Material

Supplementary Table 1. The vestibular reflex tests performed in each center. (vHIT = video Head Impulse Test, cVEMP = cervical vestibular evoked myogenic potential, oVEMP = ocular vestibular evoked myogenic potential).

	**Caloric test**	**Torsion swing test**		**vHIT**			**cVEMP**	**oVEMP**
		0.1 Hz	0.05 Hz	Horizontal	Anterior	Posterior		
Center 1 (n=50)	+	+		+	+	+	+	+
Center 2 (n=58)	+		+	+	+	+	+	
Center 3 (n=65)	+			+				

Supplementary Table 2. Overview of etiology of bilateral vestibulopathy (BVP) for all three centers combined. Numbers shown represent the count (n) of each etiology.

**Etiology**		**N**	
Idiopathic		61	
Genetic		29	
	DFNA9		21
	Hereditary BPV (mutation unspecified)		3
	MYO6 MYO7 gene mutation		2
	DNMT1 gene mutation		1
	MYO15A gene mutation		1
	PCDH15 gene mutation		1
Ototoxicity		28	
	Gentamicin		13
	Vancomycin		1
	Amikacin		1
	Aminoglycoside (type unspecified)		9
	Vitamin-B12 deficiency		1
	Polytoxicomania		1
	Hemodialysis		1
	Chemotherapy Non-Hodgkin lymphoma (type unspecified)		1
Infectious		21	
	Meningitis		7
	Vestibular neuritis		6
	Recurrent bilateral otitis media		3
	Lyme disease		2
	Herpes zoster		1
	Malaria		1
	Encephalitis		1
Menière's Disease		12	
Trauma		6	
	Traumatic craniocerebral injury		5
	Post-radiotherapy		1
Auto-immune		5	
	Hypothyroidism		2
	Rheumatic Disease		1
	Acquired Ichthyosis vulgaris		1
	Unspecified auto-immune disorder		1
Neurodegenerative		5	
	CANVAS		5
Congenital		4	
	CHARGE		1
	Syndrome unspecified		3
Mixed		2	
	Unilateral vestibular schwannoma in combination with unilateral idiopathic cause		2

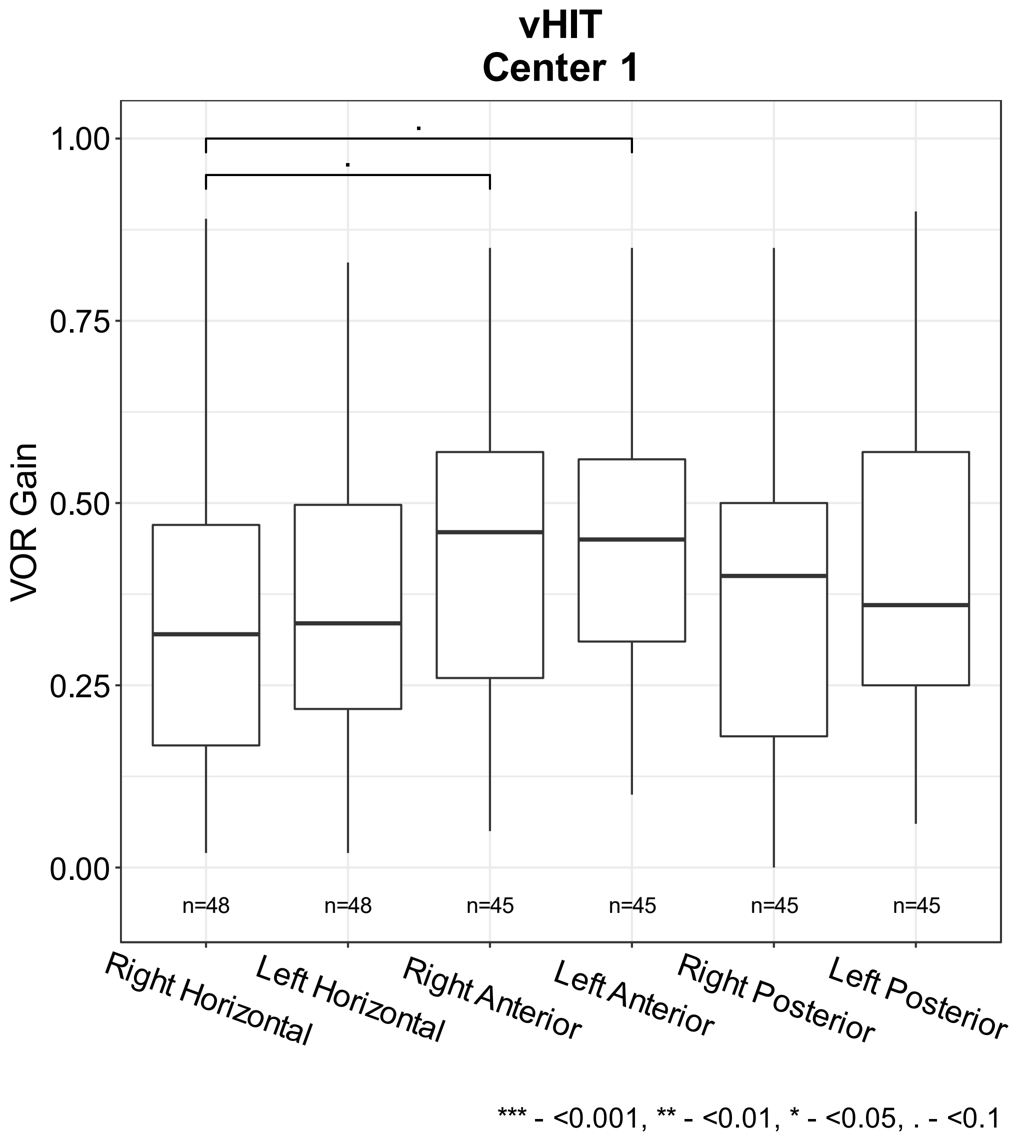

Supplementary Figure 1. Vestibular Ocular Reflex (VOR) gain of all six semi-circular canals measured with the video Head Impulse Test (vHIT) in center 1. Each box plot represents the 25 to 75 percentiles and bold black lines the median.

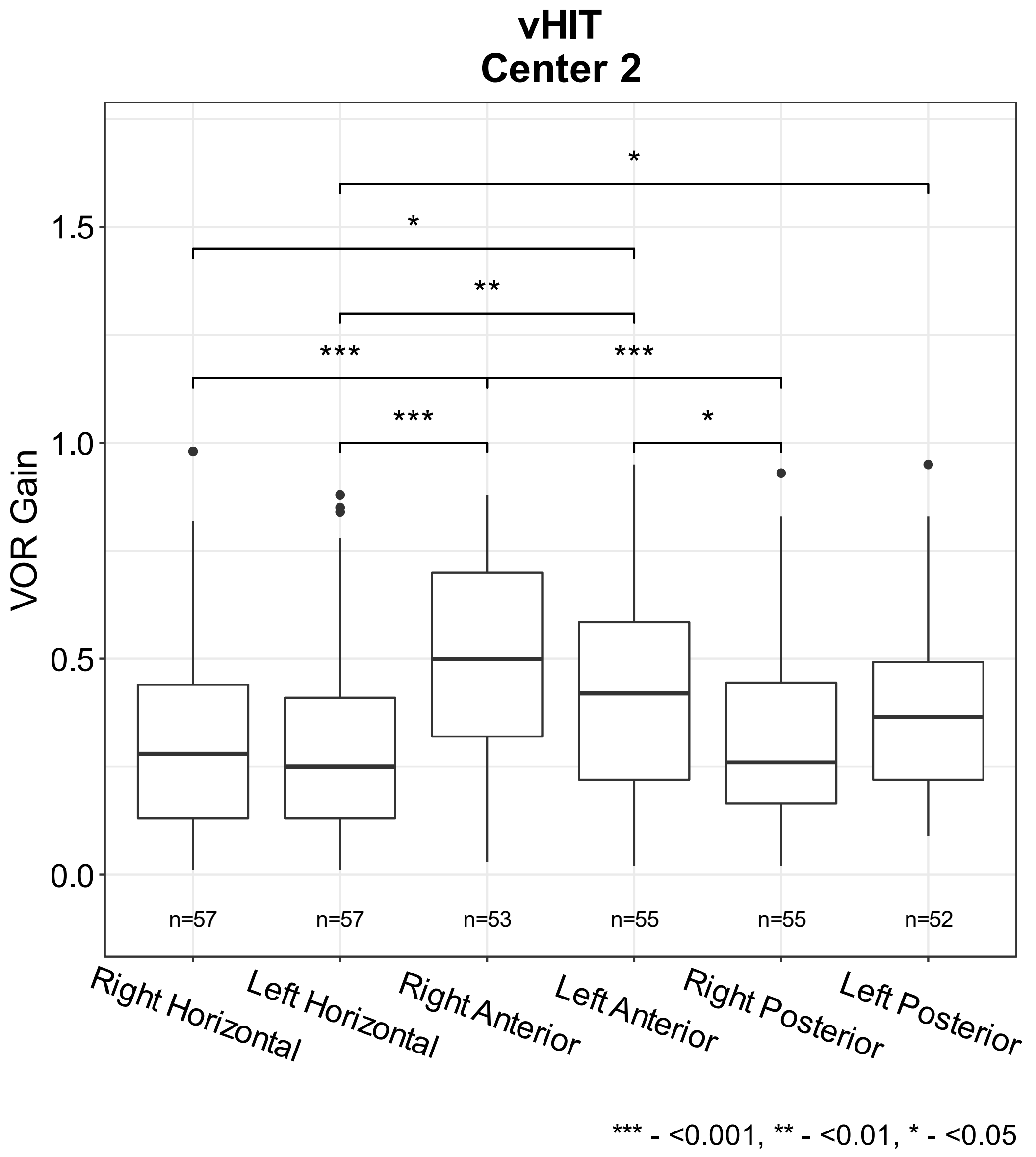

Supplementary Figure 2. Vestibular Ocular Reflex (VOR) gain of all six semi-circular canals measured with video Head Impulse Test (vHIT) for center 2. Each box plot represents the 25 to 75 percentiles, bold black lines the median, dots the outliers and asterisks (*) illustrate statistically significant differences.

Supplementary Table 3. Statistical results from pairwise comparisons between the six semi-circular canals of all three centers combined and per center separately (center 1 and 2) using post-hoc Dunn test with Benjamini Hochberg correction for multiple testing. Significant p-values are shown in bold font. (LA = Left Anterior, RA = Right Anterior, LH = Left Horizontal, RH = Right Horizontal, LP = Left Posterior, RP = Right Posterior)

	**All 3 centers**		**Center 1**		**Center 2**	
	**Z**	**p-value**	**Z**	**p-value**	**Z**	**p-value**
LA - LH	3,520	**0,002**	1,845	0,325	3,365	**0,003**
LA - LP	1,244	0,291	1,110	0,445	0,622	0,572
LH - LP	-2,101	0,067	-0,718	0,591	-2,689	**0,018**
LA - RA	-1,053	0,366	0,302	0,817	-1,611	0,146
LH - RA	-4,671	**<0.001**	-1,539	0,31	-4,958	**<0.001**
LP - RA	-2,282	**0,048**	-0,808	0,571	-2,205	0,052
LA - RH	3,235	**0,004**	2,852	0,065	2,703	**0,021**
LH - RH	-0,328	0,857	1,023	0,459	-0,668	0,582
LP - RH	1,819	0,115	1,724	0,318	2,037	0,069
RA - RH	4,388	**<0.001**	2,545	0,082	4,302	**<0.001**
LA - RP	2,895	**0,009**	1,664	0,288	2,408	**0,034**
LH - RP	-0,29	0,827	-0,155	0,877	-0,935	0,437
LP - RP	1,629	0,155	0,554	0,669	1,752	0,120
RA - RP	3,932	**<0.001**	1,362	0,371	3,997	**<0.001**
RH - RP	-0,005	0,996	-1,161	0,461	-0,273	0,785

Supplementary Table 4. Kruskal-Wallis H test results for comparison of median vestibular test results for caloric test, torsion swing test and 3D-video Head Impulse Test between etiologies. Significant p-values are shown in bold font. (X^2 = Chi-squared statistic, Df = Degrees of freedom, vHIT = video Head Impulse Test)

		**X^2**	**Df**	**p-value**
Etiology – Caloric test	center 1	4.685	6	0.585
	center 2	6.557	8	0.585
	center 3	11.400	7	0.122
Etiology – Torsion swing test	center 1	3.213	6	0.782
	center 2	21.830	9	**0.009***
Etiology – vHIT Anterior	All 3 centers	14.434	9	0.103
	center 1	2.1420	6	0.906
	center 2	17.234	9	**0.045***
Etiology – vHIT Horizontal	All 3 centers	32.389	9	**<0.001***
	center 1	5.623	6	0.467
	center 2	17.387	9	**0.043***
	center 3	24.491	7	**<0.001***
Etiology – vHIT Posterior	All 3 centers	16.047	9	0.066
	center 1	4.564	6	0.601
	center 2	17.670	9	**0.039***

* Significant results from Kruskal-Wallis H test were further analyzed with pairwise comparisons using post-hoc Dunn test with Benjamini Hochberg correction for multiple testing, shown in table 4.

Supplementary Table 5. Statistical results from pairwise comparisons of torsion swing test results from center 2, horizontal vHIT results from all three centers, 3D vHIT results from center 2 and horizontal vHIT results from center 3, between different etiologies using post-hoc Dunn test with Benjamini Hochberg correction for multiple testing. Significant p-values are shown in bold font. (vHIT = video Head Impulse Test)

	**Torsion swing test center 2**		**Horizontal vHIT all 3 centers**		**Anterior vHIT center 2**		**Horizontal vHIT center 2**		**Posterior vHIT center 2**		**Horizontal vHIT center 3**	
	Z	p-value	Z	p-value	Z	p-value	Z	p-value	Z	p-value	Z	p-value
Autoimmune - Congenital	-1,651	0,371	-0,037	1,000	0,412	1,000	-0,064	0,971	-0,513	0,804	1,204	0,376
Autoimmune - Genetic	-0,555	0,723	1,096	0,372	1,136	0,823	0,341	0,868	-1,012	0,610	-	-
Congenital - Genetic	1,730	0,418	1,145	0,378	0,568	0,987	0,430	0,883	-0,305	0,900	-	-
Autoimmune - Idiopathic	-1,232	0,491	-0,231	0,897	0,659	0,997	0,252	0,901	-0,977	0,592	1,174	0,374
Congenital - Idiopathic	1,011	0,540	-0,181	0,918	0,101	0,985	0,338	0,848	-0,282	0,898	-0,386	0,784
Genetic - Idiopathic	-2,007	0,336	-3,096	**0,022**	-1,259	0,721	-0,233	0,895	0,051	0,981	-	-
Autoimmune - Infectious	-1,726	0,380	-0,020	0,984	-0,048	0,984	-0,689	1,000	-1,680	0,299	1,620	0,268
Congenital - Infectious	0,489	0,760	0,028	1,000	-0,601	0,986	-0,603	0,983	-0,991	0,603	0,388	0,814
Genetic - Infectious	-3,183	0,065	-2,079	0,121	-3,005	0,119	-2,734	0,094	-1,792	0,299	-	-
Idiopathic - Infectious	-1,218	0,478	0,426	0,773	-1,645	0,500	-2,201	0,312	-1,671	0,266	1,161	0,362
Autoimmune - Menière's Disease	-0,959	0,524	-1,096	0,384	0,019	0,985	0,000	1,000	-1,715	0,324	0,125	0,900
Congenital - Menière's Disease	1,063	0,518	-1,052	0,388	-0,486	0,973	0,078	0,981	-1,086	0,567	-1,598	0,257
Genetic - Menière's Disease	-0,880	0,550	-3,459	**0,008**	-1,841	0,492	-0,568	0,917	-1,508	0,348	-	-
Idiopathic - Menière's Disease	0,270	0,908	-1,583	0,232	-1,024	0,860	-0,404	0,883	-1,474	0,351	-2,185	0,101
Infectious - Menière's Disease	1,067	0,559	-1,690	0,195	0,108	1,000	1,090	0,730	-0,314	0,917	-2,495	0,071
Autoimmune - Mixed	-2,126	0,503	-1,163	0,380	-0,397	0,973	-0,984	0,770	-1,886	0,296	-	-

Congenital - Mixed	-0,220	0,885	-1,133	0,373	-0,872	0,957	-0,910	0,816	-1,293	0,420	-	-
Genetic - Mixed	-2,762	0,129	-2,178	0,110	-2,221	0,296	-2,107	0,316	-1,705	0,305	-	-
Idiopathic - Mixed	-1,731	0,470	-1,234	0,376	-1,528	0,569	-1,910	0,316	-1,678	0,280	-	-
Infectious - Mixed	-1,004	0,526	-1,346	0,321	-0,557	0,963	-0,612	1,000	-0,69	0,669	-	-
Menière's Disease - Mixed	-1,639	0,350	-0,478	0,749	-0,556	0,930	-1,320	0,600	-0,362	0,897	-	-
Autoimmune - Neurodegenerative	-1,905	0,365	1,918	0,155	0,229	0,996	0,533	0,891	-1,424	0,366	2,459	0,065
Congenital - Neurodegenerative	-0,254	0,878	1,957	0,151	-0,183	0,986	0,596	0,953	-0,910	0,583	1,471	0,282
Genetic - Neurodegenerative	-2,081	0,421	1,450	0,276	-0,821	0,927	0,396	0,842	-0,948	0,572	-	-
Idiopathic - Neurodegenerative	-1,356	0,438	3,017	**0,023**	-0,349	0,962	0,469	0,871	-0,951	0,591	2,923	**0,032**
Infectious - Neurodegenerative	-0,830	0,555	2,608	**0,045**	0,355	0,985	1,404	0,555	-0,230	0,898	1,326	0,345
Menière's Disease - Neurodegenerative	-1,374	0,449	3,573	**0,016**	0,262	1,000	0,652	1,000	-0,029	0,977	3,893	**0,003**
Mixed - Neurodegenerative	-0,073	0,942	2,742	**0,039**	0,661	1,000	1,599	0,412	0,243	0,909	-	-
Autoimmune - Toxic	-1,063	0,540	0,479	0,768	0,093	0,969	0,513	0,882	-0,438	0,850	1,902	0,178
Congenital - Toxic	0,959	0,543	0,528	0,747	-0,411	0,988	0,591	0,924	0,191	0,910	0,665	0,644
Genetic - Toxic	-1,075	0,578	-1,196	0,372	-1,703	0,499	0,398	0,864	0,856	0,588	-	-
Idiopathic - Toxic	0,085	0,953	1,579	0,224	-0,892	0,986	0,506	0,862	0,789	0,605	2,250	0,098
Infectious - Toxic	0,888	0,562	0,91	0,467	0,237	1,000	1,978	0,359	1,898	0,371	0,300	0,823
Menière's Disease - Toxic	-0,147	0,925	2,483	0,059	0,106	1,000	0,726	1,000	1,805	0,320	3,428	**0,009**
Mixed - Toxic	1,508	0,395	1,722	0,191	0,650	0,967	1,969	0,315	1,976	0,433	-	-
Neurodegenerative - Toxic	1,270	0,483	-2,100	0,124	-0,187	1,000	-0,139	0,953	1,305	0,432	-1,306	0,335
Autoimmune - Trauma	-0,631	0,699	1,750	0,190	1,365	0,705	1,209	0,638	0,181	0,896	1,843	0,183

Congenital - Trauma	1,391	0,462	1,790	0,183	0,860	0,923	1,287	0,594	0,810	0,607	0,716	0,632
Genetic - Trauma	-0,262	0,892	1,215	0,374	0,660	1,000	1,706	0,360	2,002	0,509	-	-
Idiopathic - Trauma	0,858	0,550	2,913	**0,027**	1,362	0,650	1,739	0,410	1,887	0,333	1,545	0,264
Infectious - Trauma	1,636	0,327	2,464	0,056	2,439	0,331	3,183	0,065	2,970	0,134	0,416	0,825
Menière’s Disease - Trauma	0,464	0,761	3,487	**0,011**	1,903	0,513	1,710	0,393	2,681	0,110	2,726	**0,045**
Mixed - Trauma	2,055	0,359	2,617	**0,050**	2,258	0,359	2,849	0,099	2,760	0,130	-	-
Neurodegenerative - Trauma	1,702	0,363	-0,260	0,894	1,084	0,835	0,557	0,897	1,924	0,407	-0,812	0,583
Toxic - Trauma	0,611	0,696	1,917	0,146	1,798	0,464	0,984	0,813	0,876	0,591	0,218	0,858

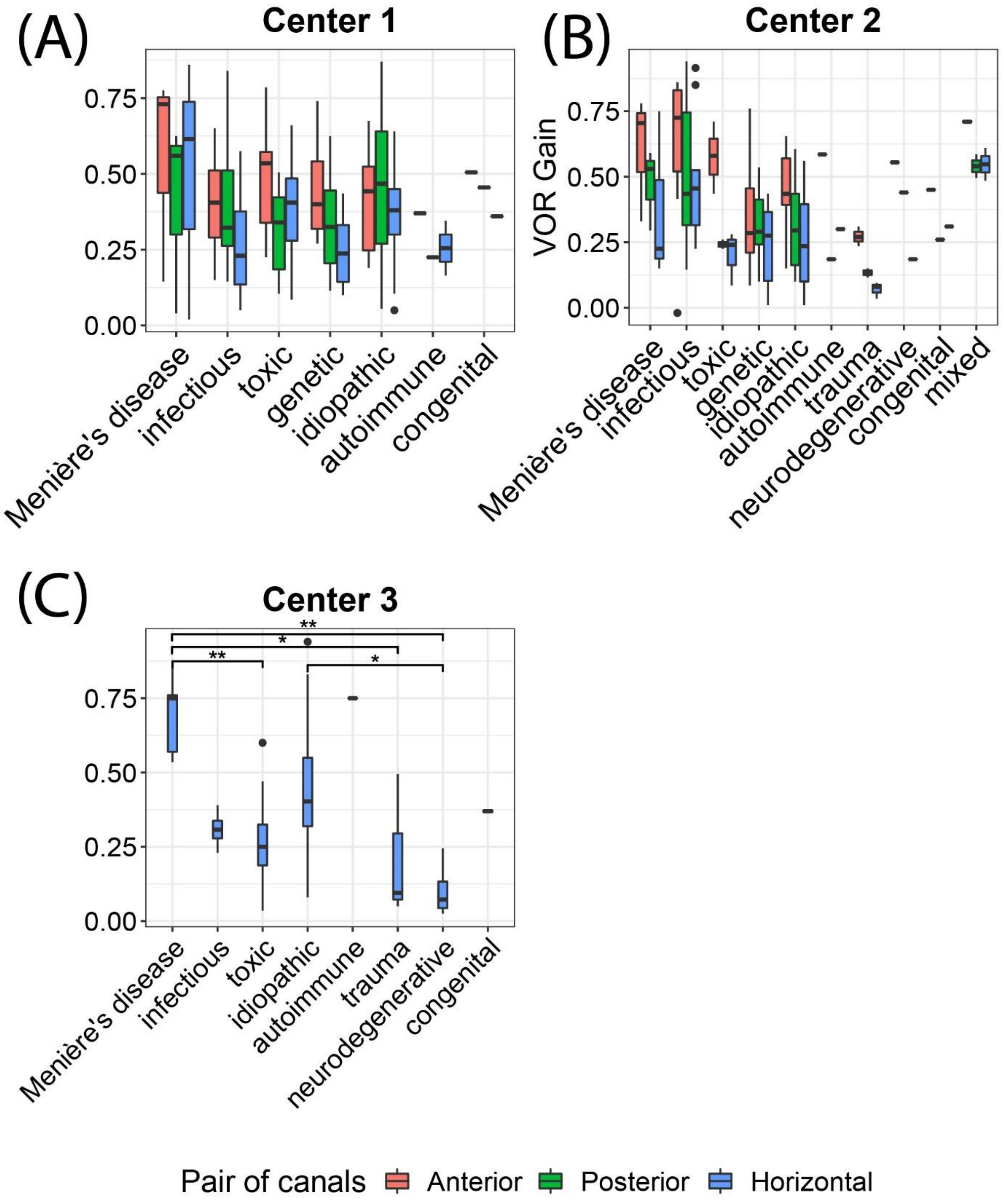

Supplementary Figure 3. Vestibular Ocular Reflex (VOR) gain per etiology for all three pairs of semi-circular canals (i.e. horizontal, anterior and posterior canals) measured with video Head Impulse Test, presented for center 1 (**A)**, center 2 (**B**) and center 3 (**C**). Each box plot represents the 25 to 75 percentiles, bold black lines the median, dots the outliers and asterisks (*) illustrate statistically significant differences (*<0.05, **<0.01).

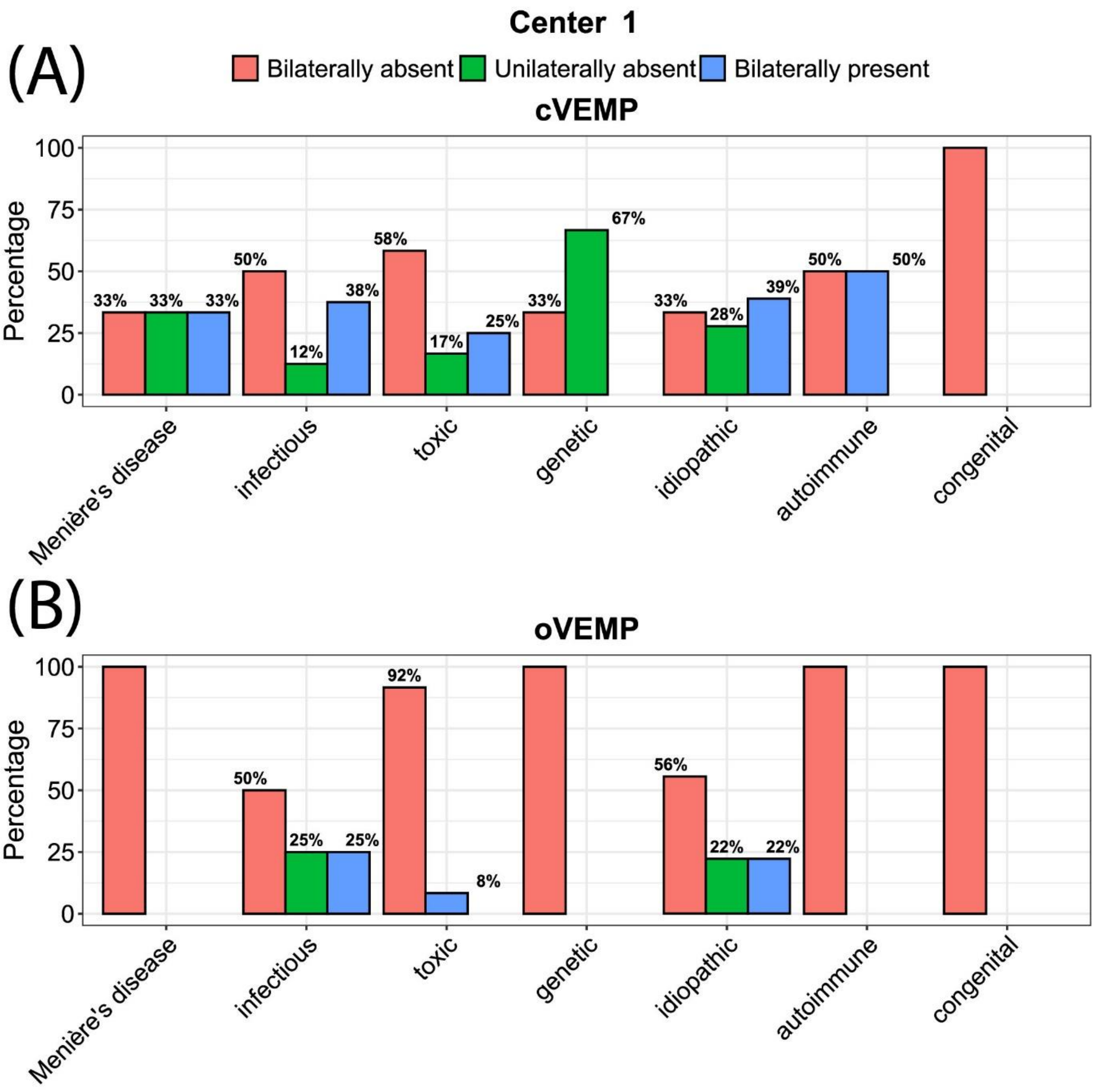

Supplementary Figure 4. The prevalence of patients in center 1 having bilaterally absent (red), unilaterally absent (green) or bilaterally present (blue) cVEMP (**A**) and oVEMP (**B**) responses.

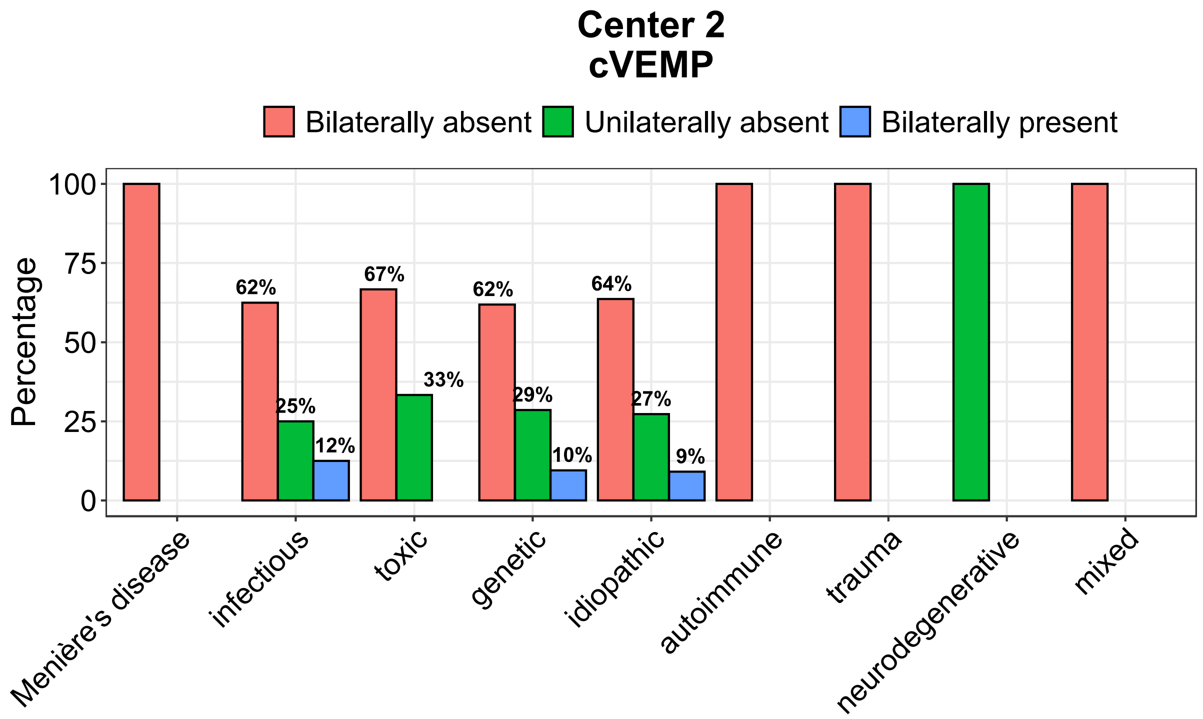

Supplementary Figure 5. The prevalence of patients in center 2 having bilaterally absent (red), unilaterally absent (green) or bilaterally present (blue) cVEMP responses.

Supplementary Table 6. Overview of all median test results with interquartile range (q1-q3) and Mann-Withney U test statistics (W and p-value) for cluster 1 "severe BVP" (n=30) and cluster 2 "moderate BVP" (n=15). Significant p-values are shown in bold font. (vHIT = video Head Impulse Test)

Cluster	Caloric test (q1-q3)	Torsion swing test (q1-q3)	Horizontal vHIT (q1-q3)	Anterior vHIT (q1-q3)	Posterior vHIT (q1-q3)
1	0,00 (0,00-0,00)	0,05 (0,02-0,11)	0,30 (0,13-0,36)	0,34 (0,23-0,46)	0,29 (0,15-0,45)
2	5,00 (3,00-7,00)	0,15 (0,09-0,27)	0,56 (0,44-0,62)	0,65 (0,54-0,70)	0,56 (0,44-0,63)
W	395	370.5	416	430	366.5
p-value	**<0.001**	**<0.001**	**<0.001**	**<0.001**	**<0.001**

Supplementary Table 7. Overview of bilaterally absent, unilaterally absent and bilaterally present cervical Vestibular Evoked Myogenic Potential (cVEMP) and ocular Vestibular Evoked Myogenic Potential (oVEMP) responses in cluster 1 "severe BVP" (n=30) and cluster 2 "moderate BVP" (n=15) with Fisher's Exact Test statistics. Significant p-values are shown in bold font.

		cVEMP (n)	oVEMP (n)
Cluster 1	Bilaterally absent	14	24
	Unilaterally absent	10	4
	Bilaterally present	6	2
Cluster 2	Bilaterally absent	6	10
	Unilaterally absent	1	1
	Bilaterally present	8	4
Fisher's Exact Test	P value	**0.044**	0.212

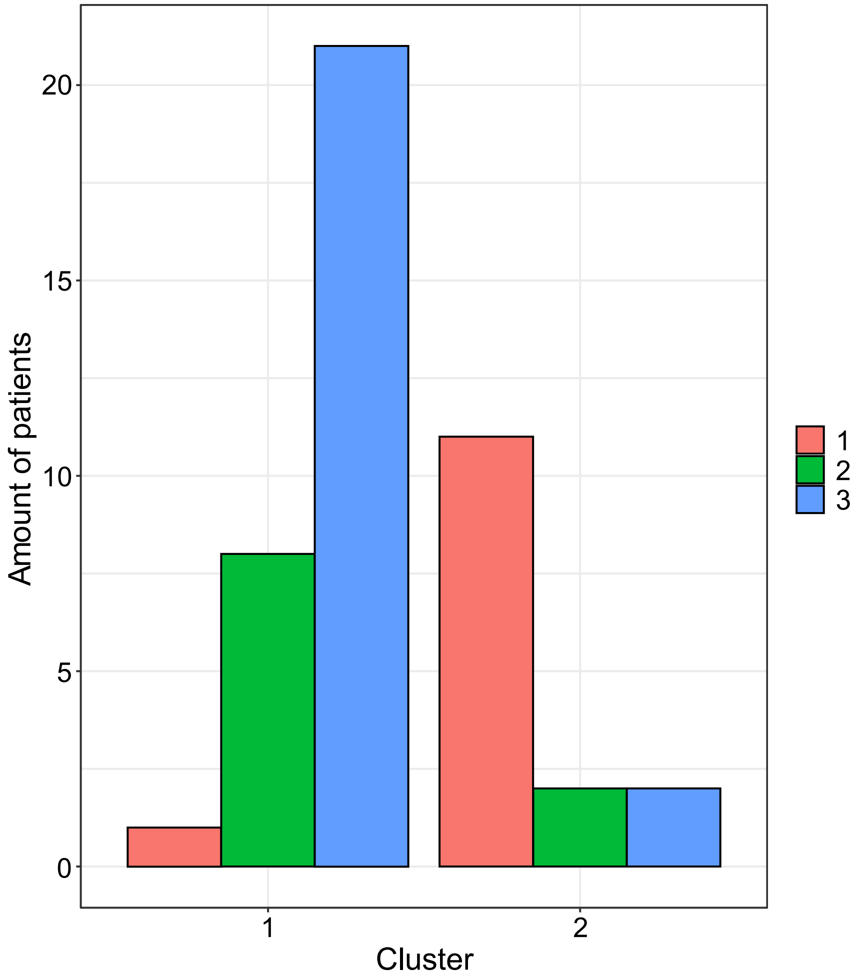

Supplementary Figure 6. The prevalence of patients meeting one (red), two (green) or three (blue) Bárány criteria per cluster (cluster 1 “severe BVP”, n=30 and cluster 2 “moderate BVP”, n=15).

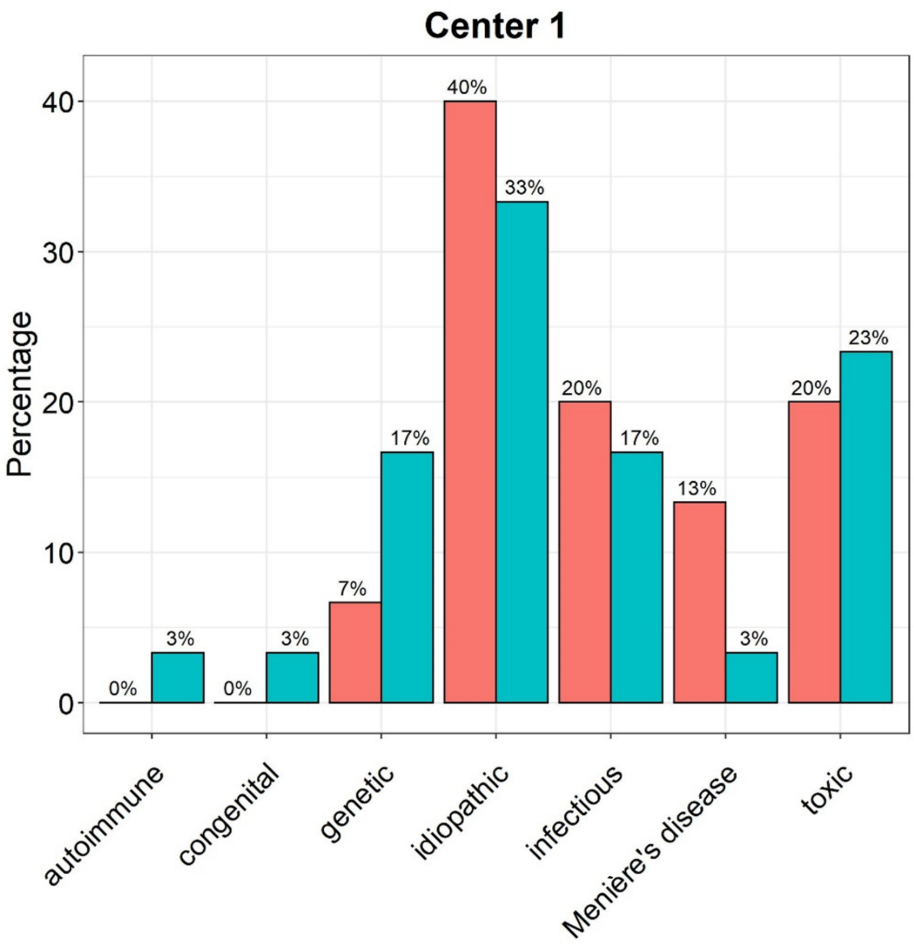

Supplementary Figure 7. The prevalence (%) of different etiologies in cluster 1 “severe BVP” (blue, n=30) and cluster 2 “moderate BVP” (red, n=15).

Supplementary Table 8. Frequency distribution of different etiologies between cluster 1 “severe BVP” (n=30) and cluster 2 “moderate BVP” (n=15) with Fisher’s Exact Test statistics.

	**Cluster 1 (n)**	**Cluster 2 (n)**
Menière’s disease	1	2
Infectious disorders	5	3
Ototoxicity	7	3
Genetic disorders	5	1
Idiopathic	10	6
Autoimmune	1	0
Congenital	1	0
Fisher’s Exact Test p-value		0.854
